# ASCL1 is a MYCN- and LMO1-dependent member of the adrenergic neuroblastoma core regulatory circuitry

**DOI:** 10.1038/s41467-019-13515-5

**Published:** 2019-12-09

**Authors:** Lu Wang, Tze King Tan, Adam D. Durbin, Mark W. Zimmerman, Brian J. Abraham, Shi Hao Tan, Phuong Cao Thi Ngoc, Nina Weichert-Leahey, Koshi Akahane, Lee N. Lawton, Jo Lynne Rokita, John M. Maris, Richard A. Young, A. Thomas Look, Takaomi Sanda

**Affiliations:** 1 Cancer Science Institute of Singapore, National University of Singapore, Singapore, 117599 Singapore; 2 Department of Medicine, Yong Loo Lin School of Medicine, National University of Singapore, Singapore, 117599 Singapore; 3 Department of Pediatric Oncology, Dana-Farber Cancer Institute, Harvard Medical School, Boston, MA 02216 USA; 4 Division of Pediatric Hematology/Oncology, Boston Children’s Hospital, Boston, MA 02215 USA; 5 The Broad Institute of MIT and Harvard, Cambridge, MA 02142 USA; 6 Whitehead Institute for Biomedical Research, Cambridge, MA 02142 USA; 7 Department of Computational Biology, St. Jude Children’s Research Hospital, Memphis, TN 38102 USA; 8 Department of Pediatrics, School of Medicine, University of Yamanashi, Chuo, 4093898 Japan; 9 Oncology Division, Children’s Hospital of Philadelphia, Philadelphia, PA 19104 USA; 10 Department of Bioinformatics and Health Informatics, Children’s Hospital of Philadelphia, Philadelphia, PA 19104 USA; 11 Department of Pediatrics, University of Pennsylvania, Philadelphia, PA 19104 USA; 12 Biology Department, MIT, Cambridge, MA 02142 USA

**Keywords:** Oncogenes, Paediatric cancer, Transcription

## Abstract

A heritable polymorphism within regulatory sequences of the *LMO1* gene is associated with its elevated expression and increased susceptibility to develop neuroblastoma, but the oncogenic pathways downstream of the LMO1 transcriptional co-regulatory protein are unknown. Our ChIP-seq and RNA-seq analyses reveal that a key gene directly regulated by LMO1 and MYCN is *ASCL1*, which encodes a basic helix-loop-helix transcription factor. Regulatory elements controlling *ASCL1* expression are bound by LMO1, MYCN and the transcription factors GATA3, HAND2, PHOX2B, TBX2 and ISL1—all members of the adrenergic (ADRN) neuroblastoma core regulatory circuitry (CRC). *ASCL1* is required for neuroblastoma cell growth and arrest of differentiation. *ASCL1* and *LMO1* directly regulate the expression of CRC genes, indicating that *ASCL1* is a member and *LMO1* is a coregulator of the ADRN neuroblastoma CRC.

## Abstract (web summary)

Polymorphisms in *LMO1* are associated with increased susceptibility to develop neuroblastoma. Here, the authors show that LMO1 directly induces the transcription factor *ASCL1*, which regulates the differentiation of neurons, demonstrating that ASCL1 is part of the adrenergic neuroblastoma core regulatory circuit.

## Introduction

Neuroblastoma is an embryonal tumor of the peripheral sympathetic nervous system, accounting for 7.5% of all cancer diagnoses in children^1–3^. The majority of children with neuroblastoma have highly invasive tumors, which are often metastatic at diagnosis. The outcome for high-risk neuroblastoma cases is very poor, with long-term survival less than 50%^3,4^. Neuroblastoma can be classified into two subtypes based on gene expression and enhancer profiles, one with a committed adrenergic (ADRN) signature and the other with mesenchymal, migratory neural crest properties (MES)^5^. Genome-wide studies revealed several recurrent molecular abnormalities in primary neuroblastoma cases, including genetic amplification of *MYCN* and mutations of *ALK*, *ATRX*, and *PTPN11*^6^. *MYCN* amplification has been used as a risk factor that is associated with a poor prognosis^3,4,7^. In addition, our recent studies have implicated *LMO1* as a major predisposition gene that functions as an oncogene in neuroblastoma^8–10^.

LMO proteins (LMO1–4) are LIM-domain-containing transcriptional co-regulatory factors that lack DNA-binding domains^11–13^. LMO proteins function as adapters to form complexes between DNA-binding proteins such as the class I basic helix-loop-helix (bHLH) proteins, class II bHLH proteins, LDB1 and GATA proteins^12,14^. *LMO2* is an oncogene that is overexpressed in T-cell acute lymphoblastic leukemia (T-ALL) due to chromosomal translocation into the vicinity of the T-cell receptor locus^12,14^. Point mutations in the noncoding elements that generate an enhancer driving overexpression of *LMO2* have also been reported^15^. *LMO1*, another member of the LMO family, is also frequently overexpressed in T-ALL due to chromosomal translocation or mutations in the enhancer^12,14^. *LMO3* is overexpressed in some T-ALL cases due to enhancer hijacking mediated by chromosomal translocation^16^. *LMO1*, *LMO2*, and *LMO3* are considered to be functionally redundant oncogenes in T-ALL^17^.

In childhood neuroblastoma, our previous genome-wide association study (GWAS) has shown that polymorphisms at the *LMO1* gene locus are strongly associated with susceptibility to tumor formation^8^. Germline single nucleotide polymorphism (SNP) risk alleles are associated with increased *LMO1* expression in neuroblastoma cell lines and primary tumors. Genetic knockdown of *LMO1* inhibits the growth of neuroblastoma cells, whereas overexpression of *LMO1* enhances proliferation in cells with low *LMO1* expression^8^. The risk allele of SNP rs2168101 G>T, which is the most highly associated variant, creates a GATA motif, and GATA3 binds at this locus^9^. This GATA3 binding is essential for the creation of a super-enhancer that drives high levels of *LMO1* expression and increases the proliferative fraction of sympathetic neuroblasts^9^. Subsequent studies showed that *LMO1* overexpression significantly accelerates the latency, penetrance, and metastatic potential of *MYCN*-induced neuroblastomas in a zebrafish transgenic model^10^. These findings implicate *LMO1* as an oncogene that collaborates with *MYCN* in neuroblastoma pathogenesis, causing arrest of neuroblast differentiation into chromaffin cells or sympathetic ganglia within the adrenal medulla, and also driving rapid neuroblast proliferation^10^. However, molecular mechanisms by which LMO1 alters transcription to drive cellular proliferation and differentiation block remain to be identified.

Recent work has suggested that a small set of transcription factors cooperate to dominate regulation of the expression program of a given cell identity through binding the majority of expressed genes/enhancers^18^. These factors form the core regulatory circuitry (CRC), which consists an interconnected autoregulatory loop whereby their expression is driven by themselves and other members of the CRC^5,19–21^. CRC members can be identified as those that are associated with top ranked regulatory elements by active histone marks such as Histone H3 lysine-27 acetylation (H3K27ac) signals^18^. As one of first examples, we have demonstrated that TAL1, GATA3, RUNX1, and MYB form the CRC in T-ALL cells^22,23^. In the ADRN subtype neuroblastoma, *PHOX2B, HAND2, TBX2, ISL1*, and *GATA3* have been implicated as CRC members^5,19–21^. Meanwhile, MYCN serves as an additional amplifier of the CRC^24^. However, the involvement of LMO1 in the neuroblastoma CRC has not been elucidated yet.

Here, we identify the transcriptional signature driven by LMO1 in combination with MYCN in neuroblastoma cells. LMO1 regulates genes in a tissue-specific manner and co-binds to the enhancers of genes regulated by the CRC transcription factors that underlie cell state in ADRN neuroblastoma. Intriguingly, LMO1 and the CRC members bind to enhancer elements and directly upregulate the *ASCL1* gene, resulting in promotion of cell growth and repression of neuronal differentiation.

## Results

### LMO1 regulates gene expression in a tumor-specific manner

**Fig. 1:**
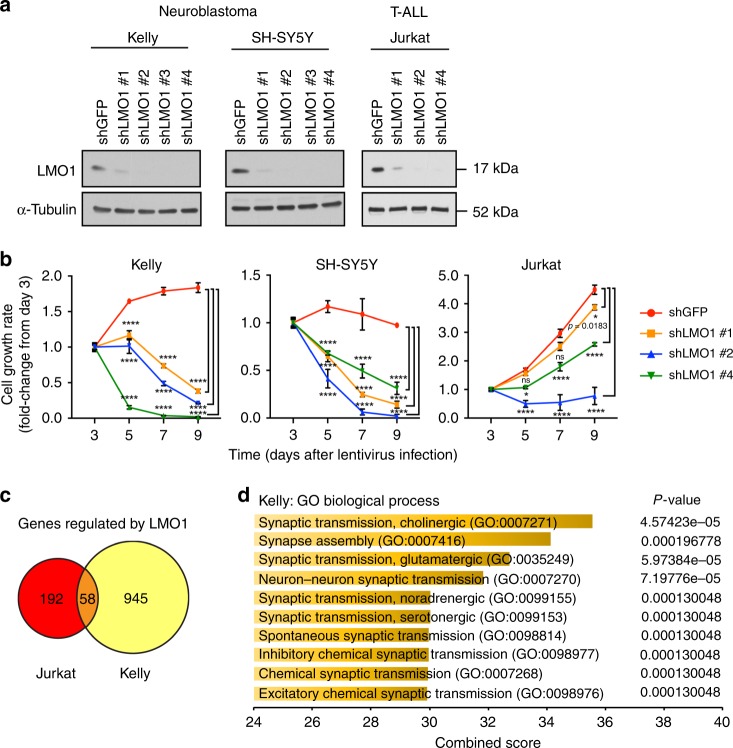
LMO1 regulates gene expressions in a tumor-specific manner. **a** Four independent shRNAs targeting *LMO1* (shLMO1 #1, 2, 3, and 4) as well as a control shRNA targeting *GFP* (shGFP) were transduced by lentivirus infection in two neuroblastoma cell lines (Kelly and SH-SY5Y) and one T-ALL cell line (Jurkat). Whole cell protein extract was harvested after 3 days of virus infection and were subjected to western blot analysis using antibodies specific for LMO1 or α-tubulin (internal control). **b** Cell viability was measured after 3, 5, 7, and 9 days of lentiviral transduction of shRNA. The growth rate (fold-change) over 9 days compared with day 3 was indicated (*n* = 3 per group). Data are represented as means ± standard deviation (SD) for technical triplicates. The *p* values by two-way ANOVA (repeated measurements) followed by Tukey's multiple comparisons post hoc test are indicated. ****p value < 0.0001. ns not significant. **c** The shRNA targeting *LMO1* or control (shGFP) was transduced into Kelly and Jurkat cells by lentiviral infection. Experiments were done in biological duplicates. Total RNAs were harvested after 3 days of infection and were subjected to RNA-seq analysis to compare gene expression profiles between two controls and two *LMO1* knockdown (KD) samples. Genes differentially expressed were selected based on the following criteria: adjusted *p* value < 0.05, log2 fold-change <−0.5 or >0.5, and TPM > 3. A Venn diagram represents the number of genes which were significantly downregulated by *LMO1* knockdown in each cell line. **d** Gene ontology analysis was performed using the gene list selected above. Top ten terms are shown with combined score. The *p* values by the Fisher exact test are indicated.

To begin to understand the role of LMO1-mediated transcriptional regulation, we first designed short-hairpin RNAs (shRNAs) to specifically knock down the expression of *LMO1* (Fig. 1a and Supplementary Fig. 1a). We chose two representative neuroblastoma cell lines (Kelly and SH-SY5Y), both of which express high levels of LMO1 protein and harbor permissive SNP alleles^8,9^. To compare gene expression profiles of LMO1-associated malignancies, we included the T-ALL cell line Jurkat, in which *LMO1* is overexpressed due to a somatically acquired point mutation in the enhancer and participates with TAL1 to promote an oncogenic transcriptional regulatory program^22^. In this setting, knockdown of *LMO1* significantly reduced cell growth in all three cell lines examined (Fig. 1b), supporting earlier findings^8^. *LMO1* expression level was positively associated with the cellular growth rate, in which *LMO1* shRNA #2 showed the greatest reduction of LMO1 expression levels (Fig. 1a) and the strongest inhibition of cell growth (Fig. 1b). The growth of neuroblastoma cell lines was more strongly inhibited by *LMO1* knockdown than that of T-ALL cells, indicating that neuroblastoma cells are highly dependent on LMO1 expression for cell growth and survival.

To analyze LMO1-regulated genes and pathways, we then performed RNA-sequencing (RNA-seq) analysis after knockdown of *LMO1* in Kelly and Jurkat cells using the shLMO1 #2. We first selected genes that were significantly downregulated or upregulated after *LMO1* knockdown compared with control samples (Supplementary Data 1). This analysis demonstrated that differentially expressed genes had limited overlap between Kelly and Jurkat cells (Fig. 1c). This is not surprising because LMO1 does not bind directly to promoter-enhancer DNAs but rather serves as a transcriptional adapter^13,25–28^. Thus, target loci of LMO1 depend on its binding to transcription factor partners that are expressed in a tissue-specific manner. Indeed, gene ontology (GO) analysis revealed that genes downregulated by *LMO1* knockdown in Kelly cells were enriched in the GO biological processes associated with synaptic transmission (Fig. 1d), while none of these biological processes were enriched in the analysis of Jurkat cells (Supplementary Fig. 1b). In contrast, different GO biological processes such as positive regulation of cell proliferation were enriched in genes differentially expressed in Jurkat cells (Supplementary Fig. 1b). These results indicate that LMO1 regulates different sets of genes in neuroblastoma and T-ALL cells.

### LMO1 co-occupies targets with the members of the ADRN CRC

Since LMO1 is known to bind to transcription factors as an adapter to elicit oncogenic effects, we next examined the genome-wide occupancy of LMO1 by chromatin immunoprecipitation sequencing (ChIP-seq) analysis in Kelly and Jurkat cells. Since a relatively high level of background signal was observed with the LMO1 antibody, we combined the results from two independent ChIP-seq experiments (Supplementary Fig. 2a). Analysis of the top DNA-binding motifs enriched at the apex of the LMO1-binding peaks indicated that these regions typically contained GATA binding motifs (Supplementary Fig. 2b). Using a co-immunoprecipitation approach, we demonstrated that FLAG-tagged LMO1 could interact with both GATA3 and LDB1 proteins when overexpressed in 293T cells (Supplementary Fig. 2c). Consistently, FLAG-tagged LMO1 could interact with endogenous GATA3 and LDB1 proteins when overexpressed in Kelly neuroblastoma cells (Supplementary Fig. 2d). This is analogous to the mechanism in T-ALL, in which LMO1 or LMO2 binds to GATA3 and LDB1^25^. Because GATA3 is a known member of the CRC that underlies cell state in both T-ALL and neuroblastoma, and is required for cell growth in both tumor types^19,22^, we next analyzed the co-occupancy of LMO1 with GATA3 by ChIP-seq.

**Fig. 2:**
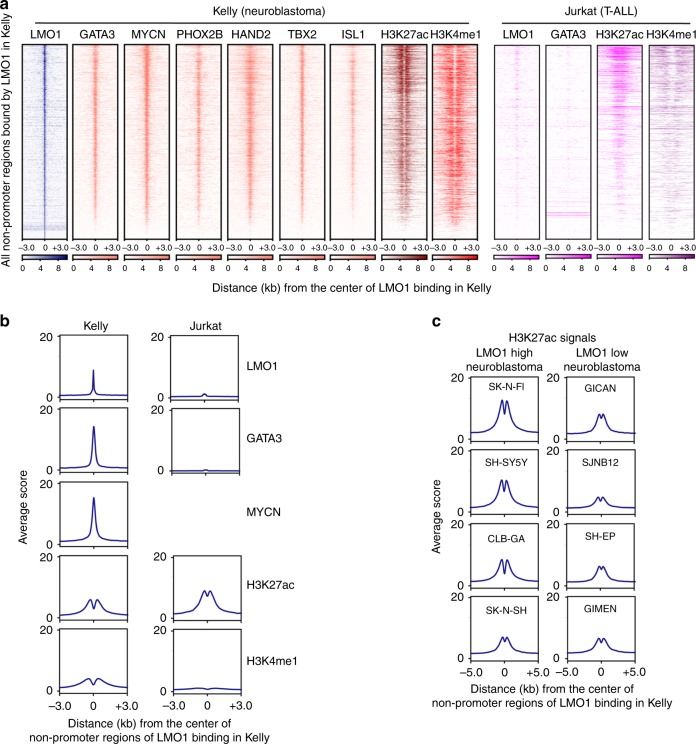
LMO1 co-occupies targets with the members of the ADRN CRC. **a** The LMO1-bound nonpromoter loci were first identified in Kelly cells. Density plots show the distribution of LMO1, GATA3, MYCN, PHOX2B, HAND2, TBX2 and ISL1, H3K27ac and H3K4me1 signals at the LMO1-bound regions (±3 kb from binding sites) in Kelly and LMO1, GATA3, H3K27ac and H3K4me1 signals at the same regions in Jurkat cells. The color scale shows the intensity of the distribution signal. **b** Metagene plots show distribution of LMO1, GATA3, MYCN, H3K27ac, and H3K4me1 signals at the LMO1-bound regions (±3 kb from binding sites) in Kelly and Jurkat cells. **c** Metagene plots show distribution of H3K27ac signals at the LMO1-bound regions (±5 kb from binding sites) in various neuroblastoma cell lines. Cell lines were classified based on the expression level of *LMO1* mRNA.

We first selected all nonpromoter regions bound by LMO1 in Kelly cells and then analyzed the occupancy of GATA3 at the same regions. We also compared the result with DNA occupancy of MYCN, which is a collaborating oncogenic transcription factor of LMO1^10,19^. Strikingly, we observed a remarkable concordance of DNA occupancy by LMO1, GATA3, and MYCN proteins in Kelly cells (Fig. 2a, left; Fig. 2b, left). In addition, LMO1 occupancy was associated with enrichment of transcription factors that have previously been shown as members of the ADRN neuroblastoma CRC, including PHOX2B, HAND2, TBX2, and ISL1^5,19–21^ (Fig. 2a, left). This result suggested that LMO1 collaborates with these factors to co-occupy the regulatory region of the same target genes. However, there was very little overlap in these LMO1-bound regions between Kelly neuroblastoma and Jurkat T-ALL cells (Fig. 2a, right; Fig. 2b, right; and Supplementary Fig. 2e), indicating that LMO1 and GATA3 bind different lineage-specific enhancers.

We next analyzed the chromatin status at LMO1 target loci in neuroblastoma cells and found that LMO1-enriched regions were frequently associated with active histone marks (H3K27ac and H3K4me1) in Kelly cells (Fig. 2a, b). We also compared H3K27ac signals at regions bound by LMO1 in Kelly cells across a panel of neuroblastoma cell lines without *MYCN* amplification that expressed different levels of *LMO1* (Fig. 2c and Supplementary Fig. 2f). This analysis revealed that cell lines that expressed high levels of *LMO1* exhibited higher levels of H3K27ac signal at LMO1-bound regions, suggesting that these cell lines have a more similar cell identity to Kelly cells, as compared with the cell lines which express low levels of *LMO1*. These results indicated that LMO1 associates with distinct genomic regions in neuroblastoma and T-ALL cells, which primarily coincide with chromatin marks of activated gene expression.

### *ASCL1* is a downstream target gene regulated by LMO1 and MYCN

We next sought to identify the individual target genes directly regulated by LMO1 in neuroblastoma cells. By integrating the results of ChIP-seq and RNA-seq analyses in Kelly cells, we predicted genes that were likely direct targets of LMO1 in which they show significant downregulation by RNA-seq after knockdown of *LMO1* and were also associated with binding sites for LMO1 and GATA3 proteins with an active histone mark (H3K27ac) by ChIP-seq (Supplementary Data 2). To confirm whether the expression of LMO1-bound genes was regulated by LMO1 in other cell line, we also performed gene expression profiling in SH-SY5Y cells by microarray after knockdown of *LMO1* using shLMO1#2. The gene set enrichment analysis (GSEA) demonstrated that many predicted LMO1 targets that were downregulated or upregulated by *LMO1* knockdown in Kelly cells were also downregulated or upregulated by *LMO1* knockdown in SH-SY5Y cells (Supplementary Fig. 3a, b). This result showed a high level of correlation in gene expression changes between two cell lines.

**Fig. 3:**
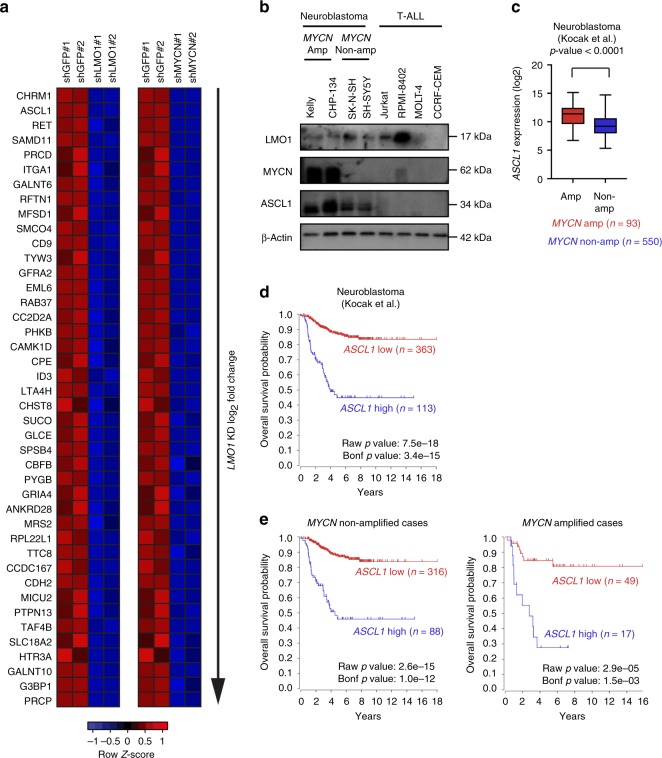
*ASCL1* a high-confidence LMO1 target in neuroblastoma cells. **a** Heatmap image represents gene expression changes of selected genes after knockdown of *LMO1* or *MYCN* in Kelly cells. The genes were ordered based on log2 fold-change values between control and *LMO1* knockdown. The color scale represents row z-scores. **b** Protein expression of LMO1, MYCN, and ASCL1 in a panel of neuroblastoma and T-ALL cell lines were analyzed by western blot. β-actin was used as an internal control. *MYCN-*amplified (amp) and nonamplified (non-amp) cell lines are indicted. **c** A total of 643 primary neuroblastoma cases in the Kocak cohort^31^ were classified into two groups: with *MYCN* amplification (*n* = 93), and without *MYCN* amplification (*n* = 550). *ASCL1* mRNA expression were shown in each group. Data are represented as box plots where the middle line indicates the median, the lower and upper hinges correspond to the first and third quartiles, the lowest datum indicates the minimum (within the 1.5 IQR of the lower quartile), and the highest datum indicates the maximum (within the 1.5 IQR of the upper quartile). The *p* values by the Mann–Whitney test are indicated. **d** The survival curve analysis for primary neuroblastoma samples in the Kocak cohort^31^ was done using the R2 database. The samples for which the prognostic data are available were classified into two groups (*ASCL1*-high and low) by the Kaplan Scan method, which calculates the optimum cutoff based on statistical testing. The raw *p* value and Bonferroni-corrected *p* value are shown. The Kaplan–Meier curve analysis for each group was done using the R2 database. **e** Primary neuroblastoma cases for which the prognostic data are available from the Kocak cohort^31^ were classified into two groups: without *MYCN* amplification (*n* = 404; left), and with *MYCN* amplification (*n* = 66; right).

Next, to identify genes most strongly dependent on MYCN for their expression among all direct targets of LMO1, we performed RNA-seq after *MYCN* knockdown (Supplementary Fig. 3c, d) and predicted genes that were directly regulated by MYCN in Kelly cells using the same criteria as for LMO1 predicted targets (Supplementary Data 2). By combining these filters, we derived a short list of genes (*n* = 42), which are displayed rank-ordered by fold-change after *LMO1* knockdown as an expression heatmap in Fig. 3a. Interestingly, the transcription factor gene with the highest fold-change in response to both *MYCN* and *LMO1* depletion was *ASCL1* (Fig. 3a). *ASCL1* encodes a bHLH transcription factor that has previously been implicated in neuronal cell development and neuroblastoma pathogenesis^28,29^. *ASCL1* expression is more highly expressed in neuroblastoma and small cell lung cancer cell lines than other cancer types in the CCLE database (Supplementary Fig. 3e). In our recent CRISPR/Cas9 screen, the *ASCL1* gene also showed selective dependency in neuroblastoma cell lines^19^. This gene was also significantly downregulated by *LMO1* knockdown in SH-SY5Y cells (Supplementary Fig. 3f). In support of these findings, neuroblastoma cell lines displayed high level expression of ASCL1 together with LMO1 by western blot analysis; however, ASCL1 was not expressed by any T-ALL cell lines examined (Fig. 3b). It is noteworthy that ASCL1 protein expression was higher in the *MYCN*-amplified neuroblastoma cell lines, Kelly and CHP-134, than in the nonamplified cell lines, SK-N-SH and its subclone SH-SY5Y (Fig. 3b), which overexpress *MYC* (*c-MYC*)^30^. Consistently, analysis of primary neuroblastoma samples demonstrated that *MYCN*-amplified cases showed a higher level of *ASCL1* expression than nonamplified cases (Fig. 3c). These results indicated that *ASCL1* is a high-confidence target gene downstream of LMO1 and MYCN in neuroblastoma cells.

### High *ASCL1* expression is associated with inferior survival

Notably, analysis using the R2 database indicated that high *ASCL1* expression was significantly associated with inferior survival of neuroblastoma patients in a cohort reported by Kocak et. al.^31^ (Fig. 3d). Two other cohorts reported by Versteeg et al.^32^ and the Neuroblastoma Research Consortium (NRC) also showed the same trend (Supplementary Fig. 3g, h), although these datasets did not show high statistical significance due to the small size of samples in cases with high *ASCL1* expression. Importantly, high *ASCL1* expression was significantly associated with inferior survival even in the absence of *MYCN* amplification (Fig. 3e). These data implicate *ASCL1* as a prognostic factor in neuroblastoma.

### LMO1, GATA3, and MYCN occupy the regulatory element of *ASCL1*

**Fig. 4:**
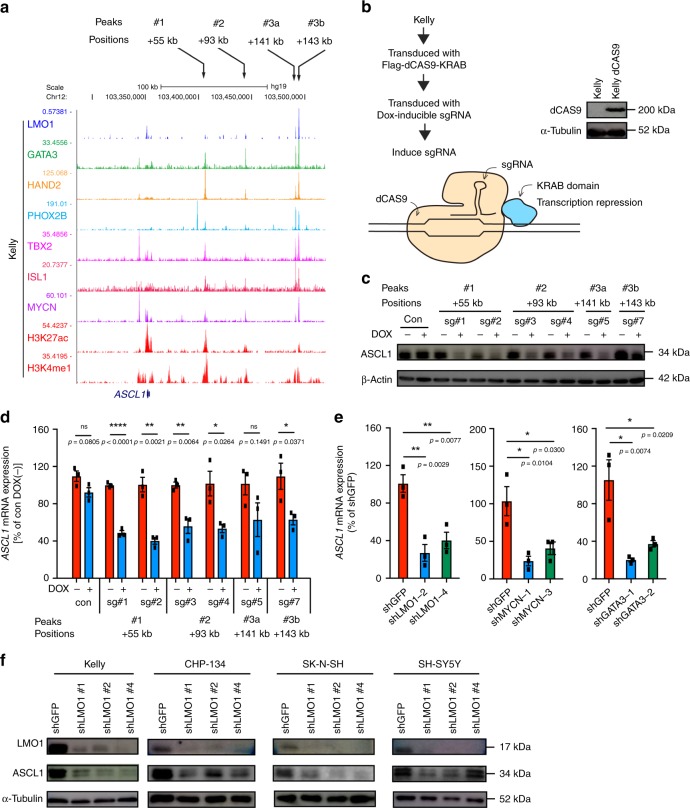
*ASCL* is directly regulated by LMO1 and MYCN in neuroblastoma cells. **a** ChIP-seq gene tracks showing the binding locations of various transcription factors at the *ASCL1* gene locus in Kelly cells. Black arrows (top) indicate regions associated with H3K27ac signals in Kelly cells (peaks 1, 2, 3a, and 3b). **b** Kelly cells was first transduced with the dCas9-KRAB protein. sgRNAs (sg#1–7) targeting each of peaks bound by LMO1 (peaks 1, 2, 3a and 3b) were then induced by the treatment with doxycycline (DOX) (right). Protein expression of dCas9 was confirmed by western blot (left). α-tubulin was used as an internal control. **c** Protein expression of ASCL1 after the induction of each sgRNA was analyzed by western blot. **d** mRNA expression of *ASCL1* after the induction of each sgRNA was analyzed by qRT-PCR. Expression was normalized to *spike-in* control RNA and shown as a percent relative to untreated control samples. Data are represented as means ± standard error of the mean (SEM) for three biological replicates. The *p* values by two-tailed unpaired t test are indicated. **e** mRNA expression of *ASCL1* after knockdown of *LMO1, MYCN*, and *GATA3* in Kelly cells were analyzed by qRT-PCR. Expression was normalized to *spike-in* control RNA and shown as a percent relative to control shGFP. Data are represented as means ± SEM for three biological replicates. The *p* value by one-way ANOVA followed by Tukey's multiple comparisons post hoc test are indicated. **f** Protein expression of LMO1 and ASCL1 were analyzed after transduction of different LMO1 shRNAs in five different neuroblastoma cell lines.

To verify that *ASCL1* is directly regulated by LMO1, we analyzed the genomic loci bound by LMO1 in neuroblastoma cells. By ChIP-seq analysis, we observed four peaks of LMO1 (+55, +93, +141, and +143 kb) located 3′ to the *ASCL1* gene and associated with H3K27ac and H3K4me1 signals in Kelly cells (Fig. 4a, bottom). Similar H3K27ac profiles were observed at the same loci in multiple ADRN subtype neuroblastoma cell lines with high level of *LMO1* expression but not in the MES subtype neuroblastoma cell lines with low level of *LMO1* expression or in the T-ALL Jurkat cell line (Supplementary Fig. 4a). Notably, only the +141 and +143 kb elements (peak 3a and 3b) were exceptionally enriched in binding of bound by LMO1, GATA3, and MYCN proteins (Fig. 4a, middle). The same loci were also co-occupied by several other transcription factor proteins (HAND2, PHOX2B, TBX2, and ISL1) that represent CRC members of the ADRN neuroblastoma^5,19–21^. We have confirmed the enrichment of GATA3 protein at the same element by ChIP-PCR in Kelly and SH-SY5Y cells (Supplementary Fig. 4b).

To further clarify whether these regions serve as distal enhancers regulating *ASCL1* expression, we engineered Kelly cells stably expressing the catalytically-dead Cas9 (dCas9) protein fused to a transcriptional repressor peptide (KRAB) (Fig. 4b). We then transduced this clone with doxycycline (DOX)-inducible single guide RNAs (sgRNAs) targeting each of these peaks. This system permits guidance to blockage of specific genomic regions by dCas9 protein coupled with repression of transcription in surrounding regions by the KRAB repressor activity. We first confirmed that dCas9 protein bound to specific sites, directed by sgRNAs, by ChIP-qPCR (Supplementary Fig. 4c). In this setting, the blockage of each enhancer region resulted in the reduction of *ASCL1* expression at the protein (Fig. 4c) and mRNA levels (Fig. 4d), indicating that each of these four enhancer regions contributes to *ASCL1* expression. Knockdown of *LMO1*, *GATA3*, and *MYCN* each separately downregulated the expression of *ASCL1* mRNA (Fig. 4e and Supplementary Fig. 4d). Downregulation of ASCL1 protein expression was observed in multiple *LMO1*-positive neuroblastoma cell lines after *LMO1* knockdown (Fig. 4f). Conversely, overexpression of *LMO1* upregulated *ASCL1* expression (Supplementary Fig. 4e, f). Taken together, these results indicated that LMO1 positively regulates the expression of *ASCL1* in concert with the CRC members GATA3 and MYCN.

### LMO1 and MYCN directly regulate the *RET* gene

**Fig. 5:**
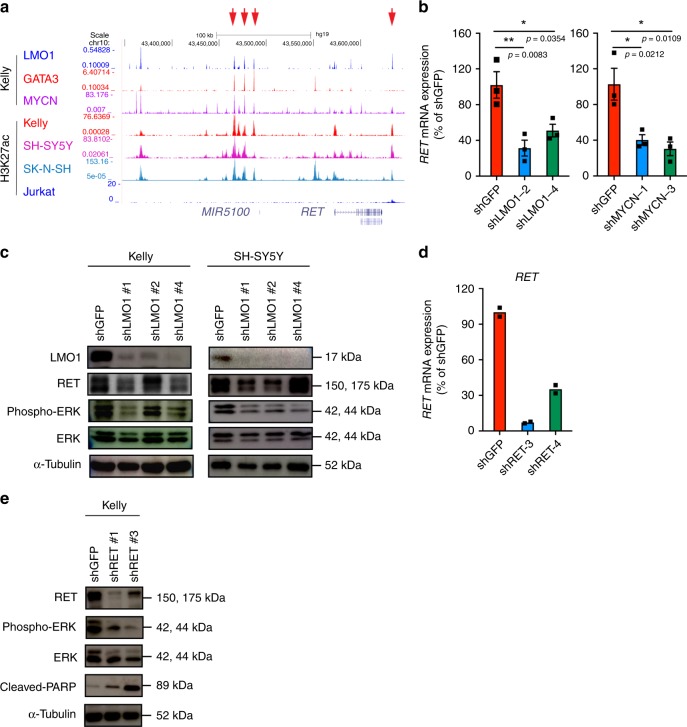
*RET* is directly regulated by LMO1 in neuroblastoma cells. **a** ChIP-seq gene tracks showing the binding locations of various transcription factors at the *RET* gene locus in Kelly cells. Red arrows (top) indicate regions associated with H3K27ac signals in Kelly cells. **b** mRNA expressions of *RET* after knockdown of *LMO*1 and *MYCN* in Kelly cells were analyzed by qRT-PCR. Data are represented as means ± SEM for three biological replicates. The *p* value by one-way ANOVA followed by Tukey's multiple comparisons post hoc test are indicated. **c** Protein expressions of RET, LMO1, p-ERK, and total ERK were analyzed after transduction of different LMO1 shRNAs in three different neuroblastoma cell lines. α-tubulin was used as an internal control. **d** The mRNA expression of *RET* after *RET* knockdown were measured by qRT-PCR. Data are represented as mean of technical duplicates. The *p* values by one-way ANOVA followed by Tukey's multiple comparisons post hoc test are indicated. **e** Protein expressions of RET, p-ERK, total ERK, and cleaved PARP were analyzed after transduction of different RET shRNAs in Kelly neuroblastoma cell line. α-tubulin was used as an internal control.

Besides *ASCL1*, we found that the receptor tyrosine kinase gene *RET* was positively regulated by LMO1 and MYCN in neuroblastoma cells (Fig. 3a). *RET* has been implicated in neuroblastoma and shown to promote cell proliferation^33,34^. In our ChIP-seq analysis, multiple binding events of LMO1, GATA3, and MYCN proteins were observed upstream of the *RET* gene locus (Fig. 5a). The expression of *RET* was downregulated after knockdown of *LMO1* at mRNA (Fig. 5b) and protein (Fig. 5c) levels in Kelly and SH-SY5Y cells. Phosphorylation of ERK1/2, which is a downstream effector of the MAP kinase pathway activated by RET kinase phosphorylation^35^, was decreased after knockdown of *RET* in the Kelly cells (Fig. 5d, e). Furthermore, knockdown of *RET* inhibited the growth and increased the fraction of Sub-G1 population in Kelly cells (Supplementary Fig. 5a, b), consistent with observations reported by others^34^. These results indicated that LMO1 and MYCN can promote cell growth and proliferation through upregulation of *RET*. However, it is noted that several LMO1-high cell lines did not express RET, whereas some LMO1-low cell lines also expressed this protein (Supplementary Fig. 5c). This suggested that LMO1 or MYCN may not be the primary determinant to induce *RET* expression, but they can positively regulate this gene when cooperating with other factors. Therefore, we highlighted ASCL1 for the downstream analysis.

### *ASCL1* is a member of the CRC of ADRN neuroblastoma

**Fig. 6:**
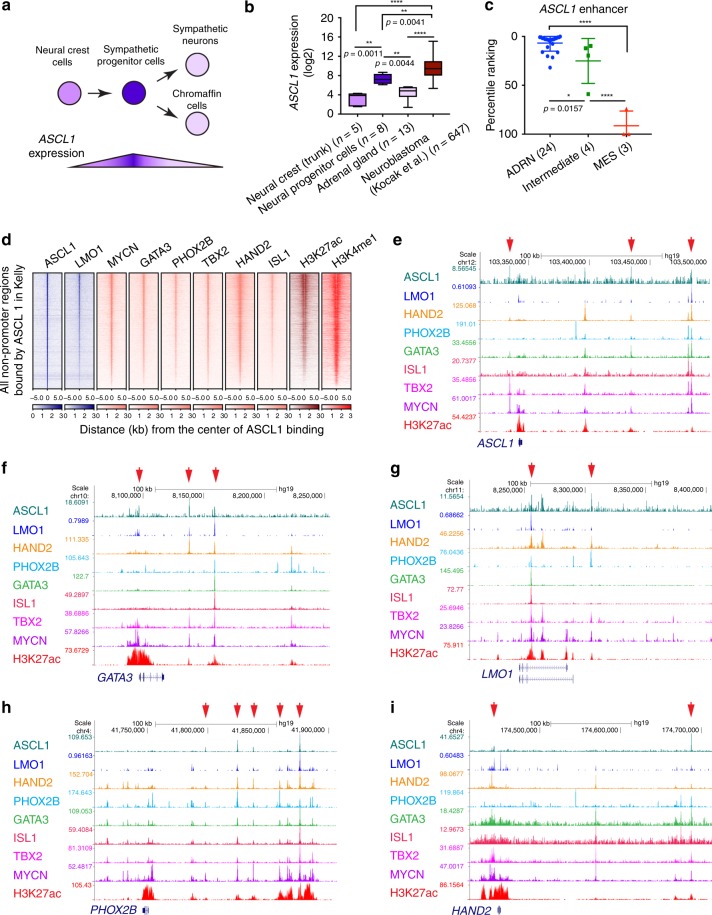
*ASCL1* is a member of CRC in the ADRN subtype of neuroblastoma. **a** Schematic image for neural differentiation and *ASCL1* expression. **b** The mRNA expression of *ASCL1* in normal neural crest, neural progenitor cells, adrenal gland, and neuroblastoma tumors (Kocak cohort^31^) were analyzed by the R2 database. The sample numbers are indicated. Data are presented as box plots where the middle line indicates the median, the lower, and upper hinges correspond to the first and third quartiles, the lowest datum indicates the minimum (within the 1.5 IQR of the lower quartile), and the highest datum indicate the maximum (within the 1.5 IQR of the upper quartile). The *p* value by one-way ANOVA followed by Tukey's multiple comparisons post hoc test are indicated in **b** and **c**. ****p value < 0.0001. **c** The dot pot for enhancer ranking in neuroblastoma cell lines (24 ADRN subtype neuroblastoma cell lines, 4 intermediate subtype neuroblastoma cell lines, 3 MES subtype neuroblastoma cell lines). Enhancers were ranked based on H3K27ac signal from high to low. Data are represented as means ± SEM. **d** The ASCL1-bound gene loci were first selected in Kelly cells. Density plots show the distribution of LMO1, MYCN, GATA3, H3K27ac, and H3K4me1 signals at the ASCL1-bound regions (±5 kb from binding sites) in Kelly cells. The color scale shows the intensity of the distribution signal. ChIP-seq gene tracks showing the binding locations of ASCL1 together with various transcription factors at the *ASCL1* (**e**), *GATA3* (**f**), *LMO1* (**g**), *PHOX2B* (**h**), and *HAND2* (**i**) gene loci in Kelly cells. Red arrows (top) indicate ASCL1-bound peak regions, which were determined by peak calling.

Since LMO1 directly regulated ASCL1 expression, we sought to understand the role of ASCL1 in mediating LMO1-driven oncogenesis. *ASCL1* has been known to be regulated in a stage-specific manner during neuronal differentiation^28,29^ (Fig. 6a). Analysis of available gene expression datasets indicated that human *ASCL1* expression was elevated in neural progenitor cells, compared with less differentiated neural crest cells or more differentiated chromaffin cells in the mature adrenal gland (Fig. 6b). Furlan et al. also reported that mouse *Ascl1* expression begins before the lineage specification that gives rise to progenitor cells that differentiate into sympathoblasts and chromaffin cells^36^. At the stage of E11.5, sympathoblasts start downregulating the expression of *Ascl1*, whereas chromaffin cells start downregulating the expression of *Ascl1* after E13.5. These observations indicated that *ASCL1* is expressed at higher levels in neural progenitor cells.

Importantly, *ASCL1* expression was high in primary neuroblastoma cells (Fig. 6b), consistent with the origin of most neuroblastomas from sympathetic nervous system progenitors. This was further supported by the fact that *ASCL1, LMO1*, and *MYCN* were each more highly expressed in the ADRN subtype of neuroblastoma cell lines corresponding to sympathetic progenitors, but less in cell lines of the MES subtype, which appear to retain features of earlier migratory neural crest cells (Supplementary Fig. 6a). ADRN cases expressed higher levels of CRC member genes (*HAND2, PHOX2B, TBX2*, and *ISL1*) than the MES subtype, whereas MES cases commonly expressed *MYC* (*c-MYC*). Consistent with these findings, analysis of our previously established neuroblastoma zebrafish models^30^ demonstrated that mRNA expression levels of the zebrafish orthologues, namely *ascl1a, lmo1, phox2bb, hand2, tbx2a*, and *isl1l*, were higher in *MYCN*-induced tumors compared with the *c-MYC*-induced tumors (Supplementary Fig. 6b). Furthermore, the *ASCL1* gene locus itself was associated with a regulatory region highly enriched for H3K27ac, indicative of an enhancer ranked in top 10% of enhancers in ADRN subtype cell lines but not in MES subtype cell lines (Fig. 6c). This result suggested that ASCL1 is possibly involved in the ADRN CRC in neuroblastoma cells.

In fact, ChIP-seq analysis demonstrated that ASCL1 frequently co-occupies regions with LMO1, MYCN and members of the ADRN CRC in Kelly cells (Fig. 6d). ASCL1 is bound to regions within regulatory elements of CRC genes at sites that were also bound by LMO1, HAND2, PHOX2B, GATA3, ISL1, TBX2, and MYCN in Kelly cells (Fig. 6e-i). The E-box motif that is recognized by type II bHLH proteins, such as ASCL1, as heterodimers with type I bHLH proteins, was significantly enriched in the ASCL1-bound regions (Supplementary Fig. 6c). These results indicated that *ASCL1* is a member of CRC in ADRN neuroblastoma.

Of note, analysis of a dataset from single cells during neural crest development by Furlan et al.^36^, showed that mouse *Ascl1* is expressed by a large fraction of the bridging cell population between Schwann cell precursors and chromaffin cells (Supplementary Fig. 6d). There was some overlap of *Phox2b, Hand2*, and *Isl1* expression with cells expressing *Ascl1*, although there was not a significant correlation between cells expressing the highest expression of *Ascl1* and expression levels of ADRN CRC genes. *Lmo1, Mycn, Tbx2*, and *Gata3* were only expressed by a small fraction of these cells. Thus, this bridging cell population is not governed by the ADRN neuroblastoma CRC. Other regulatory mechanisms may drive high levels of *Ascl1* expression in this bridging cell population.

### *ASCL1* is involved in neuroblastoma differentiation status

**Fig. 7:**
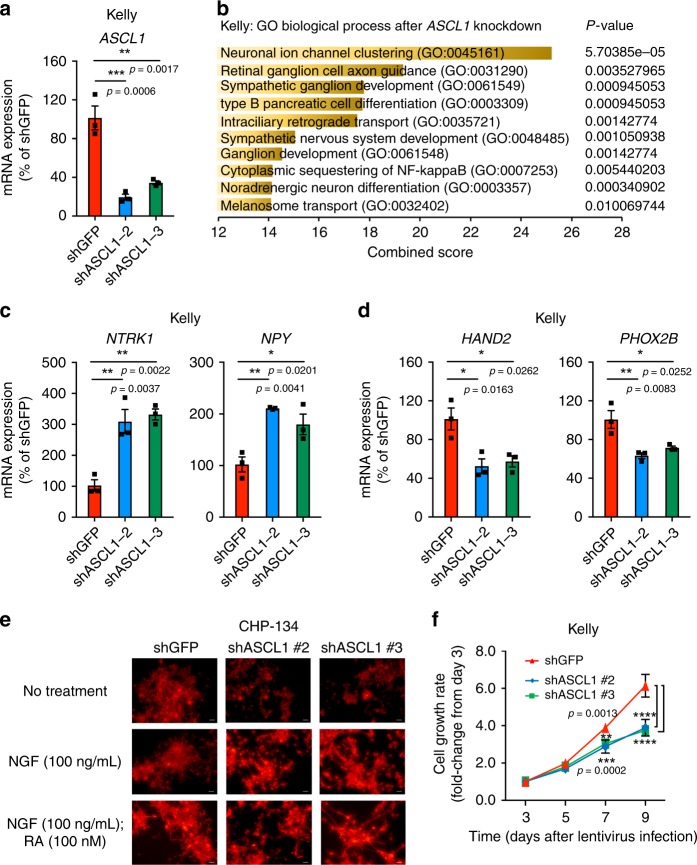
ASCL1 is required for cell growth and regulates neural differentiation status. **a** Two different shRNAs targeting *ASCL1* (#2, 3) were transduced into Kelly cells. The mRNA expression of *ASCL1* was measured by qRT-PCR, and shown as a percent relative control shGFP. Data are represented as means ± SEM for three biological replicates. The *p* value by one-way ANOVA followed by Tukey's multiple comparisons post hoc test are indicated in **a**, **c**, **d**, and **f**. ****p value < 0.0001. **b** The shRNA targeting *ASCL1* or control (*GFP*) was transduced into Kelly by lentiviral infection. Experiments were done in biological duplicates. Total RNAs were harvested after 3 days of infection and were subjected to RNA-seq analysis to compare gene expression profiles between two controls and two *ASCL1* knockdown (KD) samples. Genes differentially expressed (adjusted *p* value < 0.05, log_2_ fold-change <−0.5 and TPM > 3) were selected for gene ontology analysis. Top ten terms were shown with combined score. The *p* values by the Fisher exact test are indicated. **c**, **d** The mRNA expression of genes involved in neural differentiation and mRNA expression of CRC members were analyzed by qRT-PCR after *ASCL1* knockdown in Kelly cells. Expression was shown as a percent relative to shGFP control. Data are represented as means ± SEM for three biological replicates. **e** CHP-134 cells were treated with or without NGF (100 ng/mL) or/and ATRA (100 nM) after being transduced with shRNAs. The cells were then stained with an antineurofilament antibody, with images representative of three fields taken at ×40 original magnification. Scale bars were shown in white at bottom right, 20 μm. **f** Cell viability was measured after 3, 5, 7, and 9 days of infection in Kelly cells (*n* = 3 per group). The growth rate (fold-change) over 9 days compared with day 3 was indicated. Values represent means ± SD for technical triplicates.

Lastly, we analyzed genes and pathways controlled by ASCL1 in neuroblastoma cells. We performed an RNA-seq analysis after *ASCL1* knockdown in Kelly cells (Fig. 7a) and selected genes that were significantly downregulated after *ASCL1* knockdown compared with control samples. GO term analysis revealed that many pathways involved in the sympathetic nervous system development and noradrenergic lineage commitment were enriched for the genes downregulated by *ASCL1* knockdown (Fig. 7b).

Importantly, knockdown of *ASCL1* resulted in the increased expression of genes that are normally upregulated during the transition from neural progenitor to differentiated neuronal cells, such as *NTRK1/*TrkA and *NPY*. The result was independently validated by qRT-PCR (Fig. 7c). As a positive control, proneuronal differentiation stimuli, such as all-*trans* retinoic acid (ATRA)^37^, also resulted in upregulation of these genes (Supplementary Fig. 7a). In addition, *IGF2*, which has been reported as a target of ASCL1^38^, was upregulated after *ASCL1* knockdown (Supplementary Fig. 7b). In contrast, *ASCL1* knockdown resulted in a downregulation of several genes that are highly expressed in normal neuronal progenitor cells and are members of the neuroblastoma CRC, such as *PHOX2B* and *HAND2*. The result was independently validated by qRT-PCR (Fig. 7d). These findings indicate that ASCL1 affects the cellular differentiation status in neuroblastoma cells.

Previous studies have shown that phosphorylation also modulates the function of ASCL1^36,44^, and that the phosphorylated form of ASCL1 is associated with undifferentiated neuronal cells. Indeed, ASCL1 protein bands migrated faster after the treatment with lambda phosphatase in all cell lines examined (Supplementary Fig. 7c), suggesting that the predominant fraction is likely phosphorylated supporting the previous study by others^36,44^. Conversely, the treatment of Kelly cells with Calyculin A, a protein phosphatase inhibitor, increased this fraction (Supplementary Fig. 7d). These results suggested that the ASCL1 protein is constitutively phosphorylated in neuroblastoma cells, affecting cell differentiation status and resulting in differentiation arrest of neuroblastoma cells.

In fact, knockdown of *ASCL1* promoted axonal extension in the presence of NGF (Fig. 7e), which is a primary ligand for NTRK1. This phenotype was intensified with the presence of low dose of ATRA. *ASCL1* knockdown moderately inhibited the growth of Kelly cells (Fig. 7f), which was accompanied with a relative increase in percentage of cells in sub-G1 (Supplementary Fig. 7e). Similarly, the growth of SH-SY5Y cells, which does not possess *MYCN* amplification, was also inhibited by *ASCL1* knockdown (Supplementary Fig. 7f, g). However, *ASCL1* overexpression did not rescue the cell growth inhibition caused by *LMO1* knockdown (Supplementary Fig. 7h–j), indicating that the effects of the CRC in maintaining neuroblastoma cell growth and survival depend on high levels of expression of each member of the CRC and the LMO1 CRC coregulator acting in concert.

**Fig. 8:**
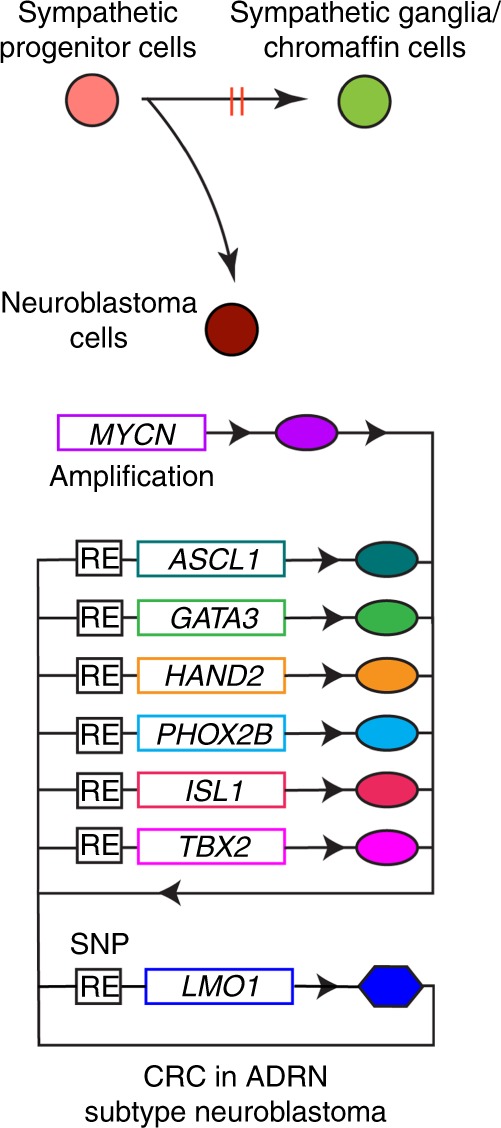
Schematic image for CRC members in ADRN type of neuroblastoma. RE regulatory element.

Taken together, our results indicate that ASCL1 contributes to the CRC with the other members to ensure viability and establish the ADRN neuroblastoma cell state (Fig. 8). In this context, LMO1 is essential for the formation of CRC and ASCL1 expression. Because LMO1 does not contain a DNA-binding domain, it does not qualify as a *bona fide* CRC transcription factor^18^. Rather, it appears to operate as an auxiliary transcriptional cofactor involved in protein–protein interactions essential for CRC activities in the regulation of cell identity and the malignant cell state in neuroblastoma cells.

## Discussion

Accumulating evidence implicates *LMO1* as a critical factor in the ADRN subtype of neuroblastoma. A large-scale neuroblastoma GWAS study demonstrated that a polymorphism within the *LMO1* gene locus is strongly associated with the susceptibility of children to develop neuroblastoma^8^. The crucial SNP mediating this neuroblastoma phenotype consists of an ancestral G permissive allele that forms a GATA DNA-binding motif^9^. GATA3 then participates with other CRC transcription factors to generate a potent enhancer that activates *LMO1* gene expression. The protective T allele at this SNP, which appeared during evolution in human populations, does not support GATA3 binding. As a member of the ADRN CRC, GATA3 binding is essential for the super-enhancer to form that drives high levels of *LMO1* expression. Because LMO1 lacks a DNA-binding domain, it cannot be a member of the ADRN CRC in neuroblastoma, but rather it serves as an essential transcriptional regulatory cofactor for formation of the autoregulatory loop that comprises the CRC, and the absence of *LMO1* expression leads to decreased levels of each of the ADRN CRC transcription factors.

Importantly, here we provide an insight into the mechanisms by which *LMO1* contributes to the ADRN neuroblastoma pathogenesis. Our study shows that LMO1 is essential for proper expression of *ASCL1*, a newly identified ADRN CRC member. It is noteworthy that we and other groups independently identified PHOX2B and HAND2 as key components of the CRC in ADRN neuroblastoma cases^5,19,21^. Our current results demonstrate that ASCL1 binds to the associated enhancers and regulates the expression of CRC members—*PHOX2B, HAND2, GATA3, TBX2*, and *ISL1*—in concert with LMO1 and MYCN. Each of these transcription factors are highly expressed in the ADRN subtype of neuroblastoma as compared with the MES subtype. ASCL1 co-binds with LMO1 at the loci of binding of the CRC transcriptional protein complex within regulatory elements of each CRC gene. This mechanism likely relates to the established proclivity of LMO1 to participate in transcriptional complexes containing the class II bHLH proteins (TAL1, TAL2, or LYL1) observed in T-ALL^13^. Given that ASCL1 is also a selective neuroblastoma dependency gene whose locus is bound by the CRC delineated by our prior work^19^, and whose product binds coordinately with the ADRN CRC members to enhancers controlling each of the CRC members and genes of the extended regulatory network, we propose that ASCL1 is also an essential transcription factor and member of the feed-forward autoregulatory loop that comprises the CRC in the ADRN subtype of neuroblastoma.

In this context, MYCN further promotes gene expression program induced by the ADRN CRC in neuroblastoma cells. A recent study suggests that “enhancer invasion” shapes MYCN-dependent transcriptional upregulation in neuroblastomas with *MYCN* gene amplification^24^. Loss of MYCN leads to a global reduction in transcription, which is more evident at the MYCN target genes with the highest enhancer occupancy. In particular, tissue-specific enhancers define highly tumor-specific MYCN target genes. In fact, we observed that MYCN frequently co-occupies the enhancers of target genes with LMO1, including those of CRC members, which characterize the ADRN subtype neuroblastoma. Hence, when expressed at high levels, MYCN serves as an “enhancer invader” that reinforces the gene expression program of the entire adrenergic CRC, including LMO1 as a coregulator of this CRC.

During normal peripheral nervous system development, *ASCL1* is expressed in sympathetic progenitor cells and extinguished when cells become differentiated into neurons or chromaffin cells^28,29^. Narayanan et. al. reported that ASCL1 not only promotes the acquisition of a proneural phenotype in glioblastoma cells but also represses mesenchymal features^39^. A similar relationship may exist in neuroblastoma cells, in which the more neural crest-like mesenchymal cells do not express *ASCL1*, and *ASCL1* expression is specifically upregulated as cells differentiate along the sympathetic lineage and express members of the ADRN CRC. In fact, our study demonstrated that ASCL1 can positively regulate CRC genes that are expressed in neuronal progenitor cells such as *PHOX2B* and *HAND2* and also negatively regulates genes expressed in more differentiated cells such as *NTRK1* and *NPY*. Thus, high levels of *ASCL1* expression, along with LMO1 and MYCN, helps to mediate a differentiation arrest at the neural progenitor stage, which is an essential component of childhood neuroblastoma. Of note, comparisons shown in Supplementary Fig. 6d argue that, although many of the cells bridging between Schwann cell precursors and chromaffin cells express high levels of *Ascl1*, this expression is not regulated by the CRC of the ADRN subset of human neuroblastoma. This finding is compatible with these bridging cells contributing to neuroblastoma through a different developmental CRC.

In this study, we also compared genomic loci bound by LMO1 and gene expression profiles after *LMO1* knockdown between neuroblastoma and T-ALL cells. Although LMO1 frequently co-occupies target loci with GATA3 in both neuroblastoma and T-ALL cells, there was little overlap among the regulated enhancers between these two cell types. Similarly, genes and pathways regulated by *LMO1* knockdown in neuroblastoma cells were distinct from those in T-ALL cells. These results indicate that LMO1 regulates gene expression in a tissue/tumor-type specific manner, consistent with the fact that it acts in cooperation with type II bHLH proteins, which are known to play key roles in establishing cell lineage^13,25–28^. Because LMO1 alone cannot directly bind to DNA, the target sites of LMO1 largely depend on the binding of its protein–protein interaction partners, which are DNA-binding transcription factors expressed in specific tissues, whose binding may also be also affected by epigenetic alterations and chromatin status. It is intriguing that LMO1 participates as a cofactor in CRCs containing GATA3 in both T-ALL and neuroblastoma; however, the remainder of the CRC of T-ALL and neuroblastoma remain completely distinct. The extensive studies of the roles of LMO proteins in T-ALL and the control of hematopoiesis^13^ provide a model for future studies to define the transcriptional complexes that mediate gene expression through the ADRN CRC in neuroblastoma.

Taken together, our study shows that ASCL1 is an integral member of the neuroblastoma CRC, essential for the formation of the feed-forward autoregulatory loop that drives the oncogenic transformation in concert with MYCN. In this context, LMO1 is a critical cofactor of the CRC. Our data provide evidence that the neuroblastoma CRC, operating through an LMO1-ASCL1 axis, not only promotes the growth and survival of malignant neuroblasts, but also blocks the differentiation of sympathetic progenitor cells.

## Methods

### Cell culture and reagents

All neuroblastoma cell lines (Kelly, SH-SY5Y, CHP-134, and SK-N-SH) and T-ALL cell lines (Jurkat, RPMI-8402, MOLT-4, and CCRF-CEM) were cultured in RPMI-1640 medium (Biowest) supplemented with 10% FBS (Biowest) and 1% penicillin/streptomycin (Thermo). All cell lines were confirmed by DNA fingerprinting using the PowerPlex 1.2 system (Promega, Madison, WI, USA) and were regularly tested for mycoplasma contamination. 293T cells were cultured in DMEM medium (Biowest) supplemented with 10% FBS (Biowest) and 1% penicillin/streptomycin (Thermo). Cells were incubated in 37 °C with 5% CO_2_. ATRA, NGF, and DOX hydrochloride were purchased from Sigma and dissolved in DMSO.

### Gene knockdown

The shRNA sequences were designed according to the RNA Consortium’s recommendation (http://www.broadinstitute.org/rnai/trc) and cloned into the lentiviral vector pLKO.1-puro at the Age I and EcoR I digestion site. Each individual shRNA vector (500 ng) was co-transfected into 293T cells with the packaging plasmids pMDLg/pRRE (250 ng) and pRSV-Rev (250 ng) together with the envelope plasmid pMD2.G (250 ng) into 293T cells (200,000 cells) seeded into six-well plate 24 h before transfection, using the FuGENE 6 transfection reagent (Roche) and Opti-MEM, according to the manufacturer’s instruction. Medium was changed 24 h after transfection. Supernatants containing lentivirus was then collected after 48 and 72 h post transfection, combined, and filtered through a 0.45 mm nitrocellulose filter (Thermo). The Jurkat cells were infected with lentivirus in the presence of polybrene (8 μg/mL: Millipore) by centrifugation at 1300 rcf for 1.5 h. Neuroblastoma cells were transduced with lentivirus in the presence of polybrene (8 μg/mL: Millipore) for 2 h incubation followed by toping up fresh medium. Approximately 500,000 cells were infected with 1 mL of virus medium. The cells were then selected by the addition of puromycin (0.7 μg/mL for Jurkat cell line; 0.5 μg/mL for neuroblastoma cell lines) for at least 36 h after infection. shRNA sequences are shown in Supplementary Data 3.

### Gene overexpression

The *LMO1* cDNA (CDS region of LMO1 transcript, NM_001270428.1) was amplified and cloned into the pMSCV-IRES-GFP retrovirus vector. Retroviral vector (1000 ng) was co-transfected into 293T cells (200,000 cells) seeded into six-well plate 24 h before transfection with the packaging plasmid pMD-MLV (250 ng) and the envelope plasmid VSV-G (250 ng) using FuGENE 6 reagent (Roche) and Opti-MEM. Approximately 500,000 cells were transduced with 1 mL retrovirus media in the presence of polybrene (8 μg/ml: Millipore) for 2 h incubation followed by toping up of 1 mL fresh medium. The cells that express GFP were then sorted by flow cytometry using the BD FACSAria II (BD Biosciences).

### Cell viability assay

Neuroblastoma cells were seeded into 96-well plates (2500 cells/well) at 24 h post virus infection. Cell viability was measured after 3, 5, 7, and 9 days, based on luminescence by the Cell Titer Glo assay (Promega) using the Tecan Inifinite 200 PRO plate reader (Tecan).

### RNA extraction and quantitative reverse transcriptase PCR (qRT-PCR)

Total RNA was extracted using the NucleoSpin RNA kit (Macherey-Nagel). A total of 1000 ng of the purified RNA was reverse-transcribed into cDNA in 10 μL reaction and finally diluted to 50 μL, using the QuantiTect kit (QIAGEN) according to manufacturer guidelines. PCR mix contains 2 μL cDNA, 10 μL Power SYBR Green PCR Master Mix (Roche), and 1 μL of each forward primer and reverse primer (final concentration of 500 nM). The mRNA expression levels of the genes of interest were evaluated by ΔΔ Ct method by performing qPCR analysis the Quant Studio 3 Real-Time PCR System (Thermo Fisher Scientific) with following cycling condition: 2 min at 50 °C, 10 min at 95 °C, and 1 min at 60 °C, with ramping temperature 1.6 °C/s. Primers were tested for standard curve with serial diluted DNA to ensure efficiency between 90 and 110% and R^2^ for standard curve greater than 0.95 and single peak for melt curve to ensure the primer specificity. Primer sequences for each individual gene are shown in Supplementary Data 4.

### Protein extraction and western blot analysis

The cells were lysed in RIPA Buffer (20 mM Tris-HCl (pH 7.5), 150 mM NaCl, 1 mM Na_2_EDTA, 1 mM EGTA, 1% NP-40, 1% sodium deoxycholate, 2.5 mM sodium pyrophosphate, 1 mM beta-glycerophosphate, 1 mM Na_3_VO_4_, 1 µg/mL leupeptin) (#9806, Cell Signaling Technology) with protease inhibitors (Roche). Equal amounts of protein was diluted in Laemmli sample buffer (Bio-Rad) with 10% β-mercaptoethanol and boiled for 10 min at 95 °C. Protein samples were resolved by SDS-PAGE gel using Bio-Rad system, with running buffer (Bio-Rad), and subsequently blotted onto a PVDF membrane (Bio-Rad). Membranes were block with 5% nonfat milk for 1 h and washed with washing buffer (TBS-Tween 0.1%) following incubation with primary antibody diluted in 5% BSA (Sigma) dissolved in washing buffer, overnight at 4 °C. Incubation with secondary antibodies were performed after washing for 1 h at room temperature. HRP-labeled anti-rabbit (7074, Cell Signaling Technologies, 1:10,000 dilution) and anti-mouse (7076P2, Cell Signaling, 1:10,000 dilution) antibodies were used. Proteins were visualized by enhanced chemiluminescence (Thermo). Antibodies used for immunoblotting were as follows: LMO1 (Bethyl Laboratories, #A300314A, 1:1000 dilution), ASCL1 (Santa Cruz, 1:200 dilution, #sc-390794), MYCN (Cell Signaling Technologies, 1:1000 dilution, #84406), RET (Cell Signaling Technologies, 1:1000 dilution, #14299), ERK (Cell Signaling Technologies, 1:1000 dilution, #9102), phosphor-ERK (Cell Signaling Technologies, 1:1000 dilution, #9101), α-tubulin (Cell Signaling Technologies, 1:1000 dilution, #2144) and β-actin (Cell Signaling Technologies, 1:1000 dilution, #8457). Original uncropped images are shown in Supplementary Fig. 8.

### Blocking of genomic DNA elements

To express dCas9-KRAB protein, lentivirus was made using pLV hU6-sgRNA hUbC-dCas9-KRAB-T2a-Puro lentivirus vector (500 ng; Addgene plasmid #71236) together with packaging plasmids pMDLg/pRRE (250 ng), pRSV-Rev (250 ng) and the envelope plasmid pMD2.G (250 ng). Kelly cells were infected with virus medium using the same method mentioned in previous section. The cells were selected by puromycin (0.5 μg/mL) for 3 days. Subsequently, dCas9-KRAB expressing cells were transduced with an inducible sgRNA and a *GFP* gene. The sgRNA sequences were designed and cloned into FgH1tUTG lentivirus vector (Addgene#70183) at the BmsBI digestion site. Lentivirus were made using the vector by the method mentioned above. After 4 days of transduction of the sgRNA vector, the cells were selected by GFP signal by flow cytometry using the BD FACSAria II (BD Biosciences). The cells were then treated with 1000 ng/mL DOX to induce a sgRNA expression. The sgRNA sequences were designed using CRISPR Design Tool (http://crispr.mit.edu/) and shown in Supplementary Data 5.

### Chromatin immunoprecipitation-quantitative PCR (ChIP-qPCR)

Approximately 10 million cells were crosslinked with 1% formaldehyde (final concentration) for 10 min by inverting flasks at room temperature and quenched with 250 mM Glycine. The cells pellets were washed in PBS and then stored at −80 °C. The pellets were lysed in lysis buffer I (50 mM HEPES-KOH pH 7.5, 140 mM NaCl, 1 mM EDTA pH 8.0, 10% Glycerol, 0.5% NP-40, 0.25% Triton X-100, and complemented with protease inhibitor cocktail) for 10 min. After centrifugation, supernatant was discarded and the pellet was lysed in lysis buffer II (10 mM Tris-HCl pH 8.0, 200 mM NaCl, 1 mM EDTA pH 8.0, 0.5 mM EGTA pH 8.0, and complemented with protease inhibitor cocktail) for 10 min. After centrifugation, supernatant was discarded and the pellet was lysed in lysis buffer III (10 mM Tris-HCl, 100 mM NaCl, 1 mM EDTA pH 8.0, 0.1% SDS buffer, 1% Triton-X, and complemented with protease inhibitor cocktail) and subject to sonication. Chromatin was sheared by the Bioruptor Plus sonicator (Diagenode) at high power for 50 cycles of 30 s, with a 30-s pause between cycles. Sheared chromatin was incubated with primary antibody bound to the Dynabeads Protein G (Thermo Fisher Scientific) overnight, followed by elution and reverse cross-linking at 65 °C for 15 h. TE-buffer was added in DNA elution buffer followed by RNase treatment (0.45 mg/mL) at 37 °C for 2 h and by proteinase K treatment (0.3 mg/mL) at 55 °C for 30 min. DNA was isolated using 400 μL of the separated lower phase of the Phenol-Chloroform-isoamyl alcohol in MaXtract High Density 2 mL gel tubes (Qiagen) by centrifugation at full speed for 7 min. The aqueous layer was then transferred to a new tube and subsequently added NaCl solution (100 mM final concentration), glycogen solution (20 μg) and 800 μL 100% ethanol, and incubated at −80 °C for 1 h. DNA was precipitated by centrifuge at full speed for 20 min and washed with 75% of ethanol and pelleted. Pelleted DNA was dissolved in RNase/DNase free water. Antibodies used for ChIP-PCR were as follows: LMO1 (Bethyl Laboratories, #A300314A), Flag (Sigma, #F1804), H3K27ac (Abcam), and IgG (Santa Cruz). After the purification of DNA, quantitative PCR was performed using the Quant Studio 3 Real-Time PCR System (Thermo Fisher Scientific) with purified DNA, Power SYBR Green PCR Master Mix (Roche) and PCR primers against the genomic regions of interests. PCR primer sequences were shown in Supplementary Data 6.

### Chromatin immunoprecipitation sequencing (ChIP-seq)

For each ChIP, 10 µg of antibody was conjugated to 2 mg of M270 epoxy beads and added to 3 mL of sonicated nuclear extract. Anti-LMO1 antibody (Bethyl Laboratories, #A300314A) and anti-ASCL1 antibody (Santa Cruz, #sc-390794) were used for ChIP. Purified DNA was prepared for sequencing according to a modified version of the Solexa Genomic DNA protocol. Fragmented DNA was end repaired and subjected to linker-mediated-PCR using oligos from Illumina. Amplified fragments were purified and applied to the flow-cell using the Solexa Cluster Station fluidics device. Samples were then subjected to sequencing according to Illumina’s standard protocols. All ChIP-seq reads were mapped to the hg19 human reference genome using bowtie2 with default parameters. Duplicates were removed from aligned ChIP-seq reads using the samtools rmdup package. ChIP-seq peaks were called with MACS14 software version 1.4.2 The output bedgraph were normalized subtracting corresponding background using MACS2 bdgcmp. LMO1 peaks (*P* < 1e−4) and ASCL1 peaks (P < 1e−9) called by MACS14 were selected and filtered for nonpromoter binding peaks based on annotatePeaks from the HOMER package. Co-occurrence heatmap and metagene were plotted using deepTools version 3.1.2. Each of the signal matrix was calculated using bigwig output generated using UCSC bedGraphToBigWig of each ChIP-seq sample by deepTools computerMatrix. The signal matrix was then used to plot heatmap using deepTools plotHeatmap package^40^.

### Enhancer ranking analysis

Enhancers were defined as regions with H3K27ac ChIP-seq enrichment. H3K7ac peaks were called using MACS2 version 1.4.2 (*q*-value < 5e−2 for narrow region and *q*-value < 1e−1 for broad region) with —keep-dup = 1 and—broad. Enhancers were identified using ROSE v0.1 by merging H3K27ac peaks within 12.5 kb between each other, excluded those were fully contained within ±2 kb from TSS and ranked them along the *x*-axis based on H3K27ac enrichment plotted on the *y*-axis (https://bitbucket.org/youngcomputation/rose). All enhancers were assigned responsible to the nearest RefSeq genes. The percentile of *ASCL1* enhancer was measured by ordering the ranking index and calculating the top ranking of *ASCL1* enhancer among all other enhancers in each cell line.

### RNA-sequencing (RNA-seq)

Total RNA was extracted from control and knockdown samples using the QIAzol lysis reagent (Qiagen) and cleaned using the RNeasy kit (Qiagen). Samples were treated with the TURBO DNase (TURBO DNA-free Kit; Ambion) and cleaned using the RNeasy MinElute Cleanup kit (Qiagen). Strand-specific library construction and sequencing of ~100 M paired-end 100-bp-long reads by Illumina HiSeq or BGISEQ were performed at the BGI Biotech Solutions Co. Ltd. (Hong Kong, China). RNA-seq reads were aligned to the hg19 human reference genome using STAR 2.5.2a with outFilterMultimapNmax set to 1. Total mapped reads were quantified using htseq-count version 0.6.1, and count tables were generated based on Ensembl hg19 gene annotation gtf files. Differential expression analysis was conducted using the Bioconductor package DESeq2 version 1.12.4. Gene expression for each neuroblastoma cell lines was estimated in transcripts per million (TPM) using Kallisto software version 0.43.1. All RNA-Seq data were normalized using the Sleuth R package from Patcher lab^41^. Selected gene expression from sleuth normalized TPM were used to generate heatmap using web-based heatmap tools, heatmapper^42^. Genes differentially expressed after knockdown of each transcription factor were selected using the following criteria: adjusted *p* value < 0.05, log 2 fold-change <−0.5 or >0.5, and TPM > 3.

### GO analysis

GO analysis was performed using the Enrichr tool (http://amp.pharm.mssm.edu/Enrichr/). Combined scores were calculated by multiplying the *p* value computed by Fisher’s exact test by the *z*-score of the deviation from the expected rank.

### Immunostaining

CHP-134 cells were treated with or without NGF (100 ng/mL) or ATRA (100 nM) after transduction of shRNAs. The cells were stained with an antineurofilament antibody (Cell Signaling, #2837).

### Statistics and reproducibility

All the statistical analyses were done in GraphPad Prism software. A *p* value less than 0.05 was considered statistically significant. The details of methods used can be found in each figure legend. Experiments in Figs. 1a, b, 3b, 4b, d, e, 5b, 7a, c–f were repeated three or more times. Experiments in Figs. 4c, f, 5c–e were repeated two times. The numbers of experiments conducted in Supplementary Figures were stated in Supplementary Methods section and Supplementary Figure Legends.

### Reporting summary

Further information on research design is available in the Nature Research Reporting Summary linked to this article.

## Supplementary information

Supplementary Information

Description of Additional Supplementary Files

Supplementary Data 1

Supplementary Data 2

Supplementary Data 3

Supplementary Data 4

Supplementary Data 5

Supplementary Data 6

Supplementary Data 7

Reporting Summary

## Data Availability

ChIP-seq dataset for LMO1 and ASCL1 in Kelly cells have been deposited in the GEO database (GSE120074). ChIP-seq datasets of PHOX2B, HAND2, GATA3, MYCN, ISL1, and TBX2, H3K27ac and H3K4me1 in Kelly cells were obtained from the GEO database (GSE94824 and GSE62726)^19,43^. ChIP-seq datasets of H3K27ac in various neuroblastoma cell lines were obtained from the GEO database (GSE86672 and GSE90683)^21,44^. ChIP-seq datasets of LMO1, GATA3, H3K27ac and H3K4me1 in Jurkat cells were obtained from the GEO database (GSE94391, GSE68976, and GSE50622, GSE119439)^45–48^. RNA-seq dataset for Kelly cells after *LMO1* knockdown and *ASCL1* knockdown were deposited in the GEO database (GSE132760). Microarray dataset for SH-SY5Y cells after *LMO1* knockdown was deposited in the GEO database (GSE130747). RNA-seq dataset for Jurkat after *LMO1* knockdown has been reported by us and deposited in the GEO database (GSE97514)^48^. RNA-seq datasets for zebrafish neuroblastoma samples reported by us^30^ were obtained from the GEO database (GSE107518). RNA-seq dataset for various neuroblastoma cell lines was obtained from the GEO database (GSE90683)^21^. Single cell sequencing dataset for mouse neuronal cells was obtained from the GEO database (GSE99933)^36^. The microarray datasets for primary neuroblastoma cases reported by the Kocak et al.^31^ (GSE45547), Versteeg et al.^32^ (GSE16476) and the NRC (GSE85047) were analyzed by the R2 database (http://hgserver1.amc.nl/cgi-bin/r2/main.cgi). The cancer cell line dataset is derived from CCLE database (https://portals.broadinstitute.org/ccle). Detailed information is also shown in Supplementary Data 7. All other methods used for Supplementary Figures are described in the Supplementary Methods section.
