# Supplementary material for: ASCL1 is a MYCN- and LMO1-dependent member of the adrenergic neuroblastoma core regulatory circuitry
Source: Nat Commun. 2019 Dec 9;10:5622. doi: 10.1038/s41467-019-13515-5 (PMC6901540; doi:10.1038/s41467-019-13515-5)
Supplement: Supplementary file 1 — Supplementary Information [file 41467_2019_13515_MOESM1_ESM.pdf]

Supplementary Information

ASCL1 is a MYCN- and LMO1-dependent member of the adrenergic neuroblastoma core regulatory circuitry

Wang et al.

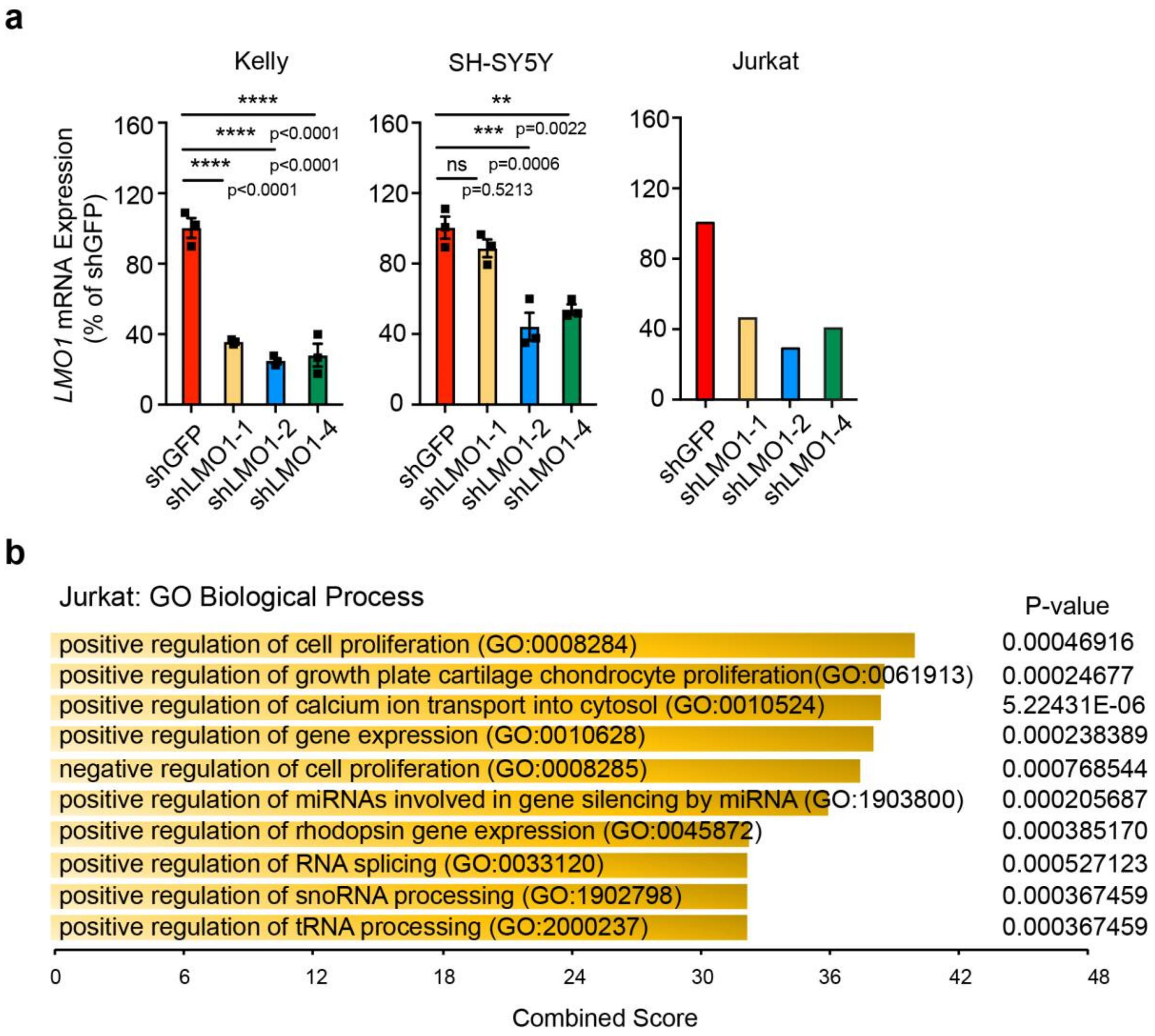

Supplementary Fig. 1: LMO1 regulates gene expressions in a tumor-specific manner. **(a)** Four different shRNAs targeting *LMO1* (#1-4) were transduced into Kelly, SH-SY5Y and Jurkat cells. The mRNA expression of *LMO1* was measured by qRT-PCR, and *GAPDH* was used as internal control. Data are represented as means ± standard error of the mean (SEM) for three biological replicates in left two panels. Data in a right panel are represented as mean of technical duplicates from one experiment. The p-values by one-way ANOVA followed by Tukey's multiple comparisons post hoc test are indicated. ****, p-value<0.0001. ns, not significant. **(b)** Gene ontology (GO) analysis was performed using the genes which were significantly downregulated by *LMO1* knockdown in Jurkat cell line. Top 10 terms were shown with combined score. The p-values by the Fisher exact test are indicated.

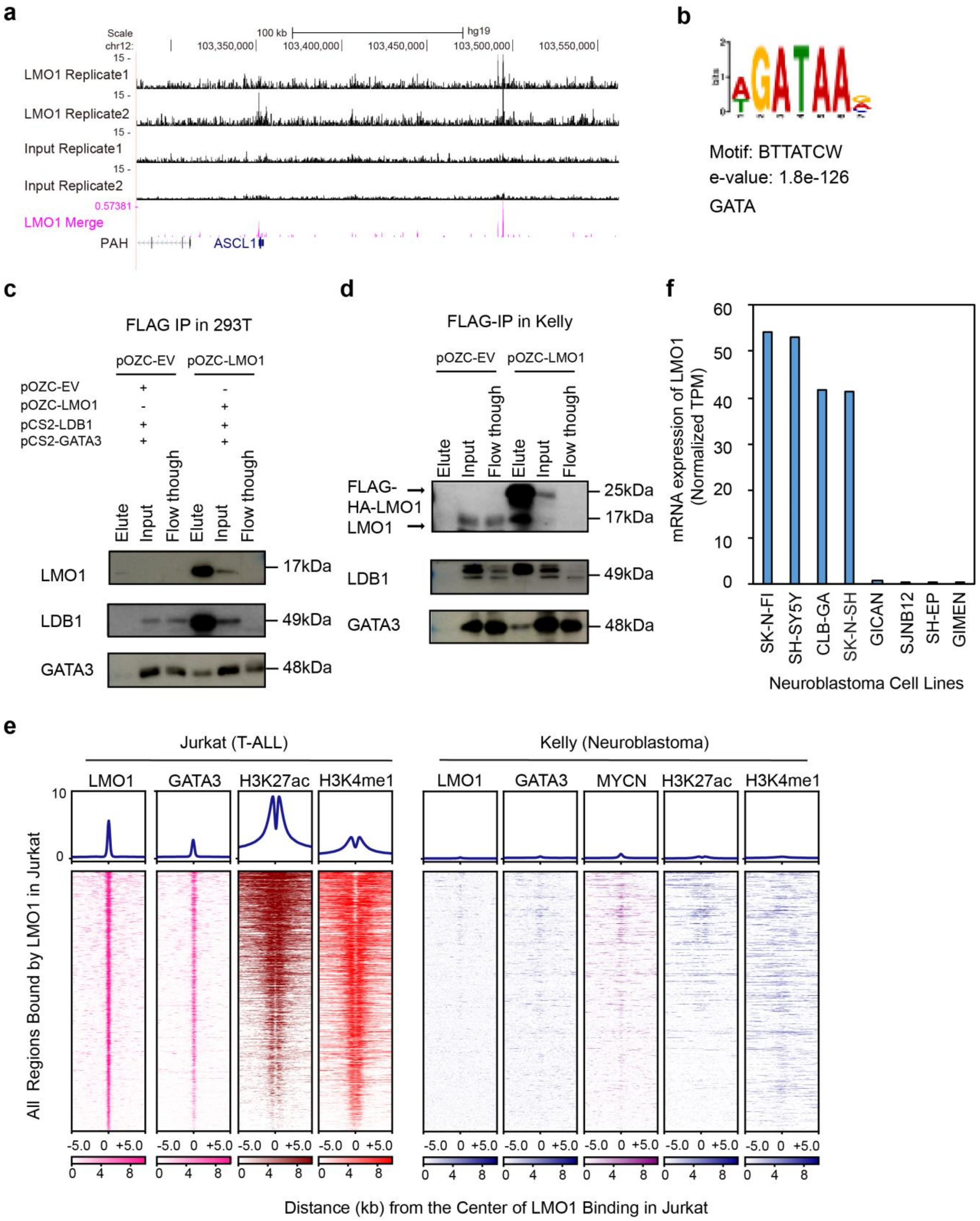

Supplementary Fig. 2: LMO1 co-occupies targets with the members of the ADRN CRC. (a) ChIP-seq gene track of two individual replicates of LMO1, input and merged LMO1 track

of two replicates**.** **(b)** DNA binding motif analysis were performed with the LMO1-bound peaks called by MACS14 (P<1e-4), 500 bps in total were retrieved from the peak summits (the highest point of each peak). Enriched motifs were analyzed using the MEME-chip package from MEME-Suite. **(c, d)** 293T cell were co-transfected with a plasmid encoding a C-terminus FLAG-HA-tagged LMO1 (pOZC-LMO1) or an empty vector (pOZC-EV) and plasmids encoding LDB1 (pCS2-LDB1) and GATA3 (pCS2-GATA3) (c). Kelly cells were transduced with pOZC-EV or pOZC-LMO1 by retrovirus infection (d). Immunoprecipitation (IP) experiments were then performed using anti-FLAG antibody. Antibodies against LMO1, LDB1 and GATA3 were used for immunoblotting. **(e)** The LMO1-bound gene loci were first selected in Jurkat cells. Density plots show the distribution of GATA3, H3K27ac and H3K4me1 signals at the LMO1-bound regions (± 5 kb from binding sites) in Jurkat and Kelly cells. Metagene plots show distribution of each transcription factor or histone mark signals at the LMO1-bound regions (± 5 kb from binding sites) in Jurkat and Kelly cells. The color scale shows the intensity of the distribution signal. **(f)** The mRNA expression of *LMO1* in different neuroblastoma cell lines were analyzed by RNA-seq (one sample for each cell line) and shown in normalized TPM.

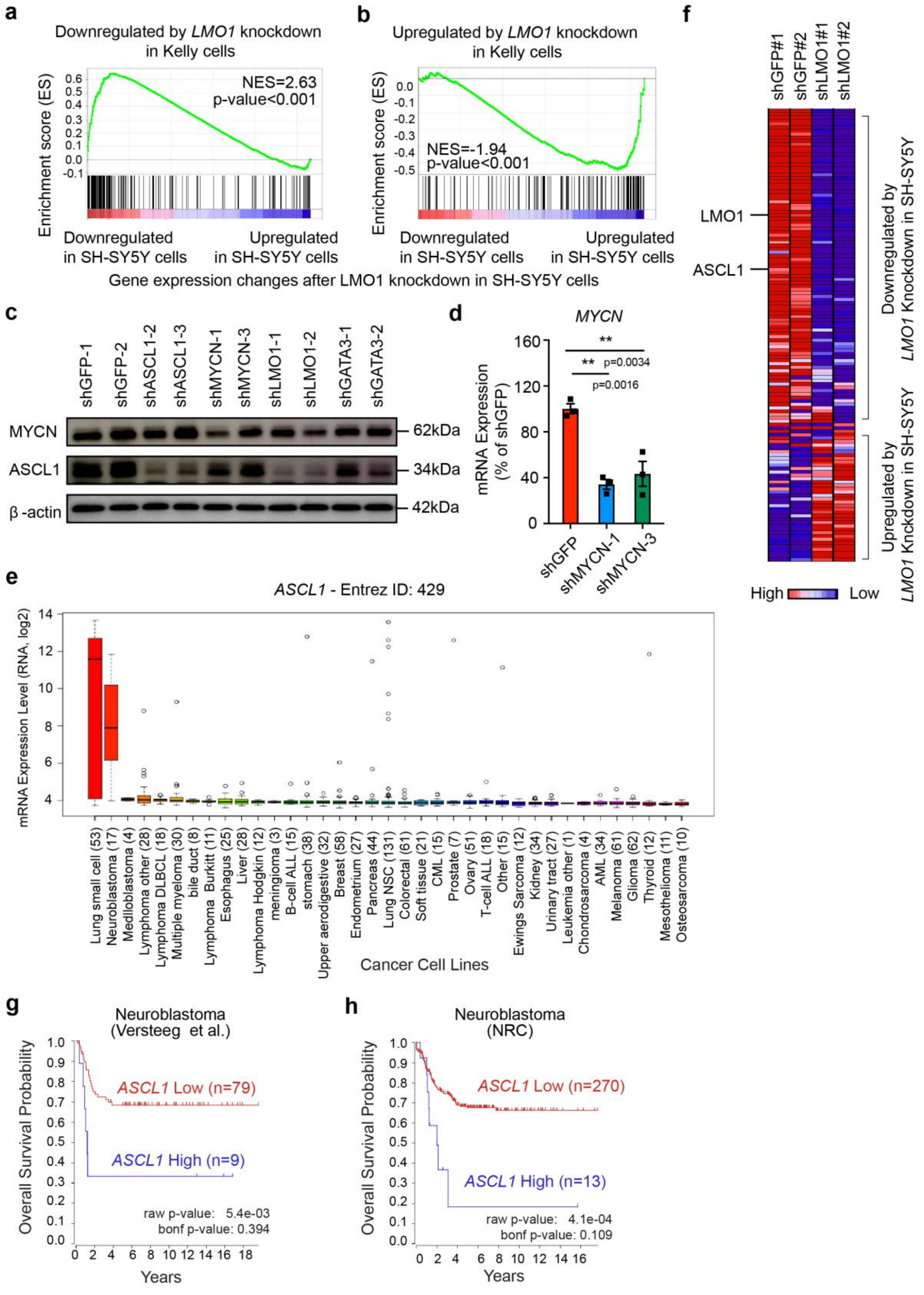

Supplementary Fig. 3: *ASCL1* a high-confidence LMO1 target in neuroblastoma cells. **(a, b)** GSEA was performed to analyze gene expression changes after *LMO1* knockdown in

SH-SY5Y cells using two gene sets that include *LMO1* target genes identified in Kelly cells:(a) downregulated by *LMO1* knockdown, and (b) upregulated by *LMO1* knockdown. GSEA plots show gene expression changes after *LMO1* knockdown in SH-SY5Y cells. Normalized enrichment scores (NES) and p-values are shown. **(c)** Two different shRNAs targeting *ASCL1, MYCN, GATA3* and *LMO1* were transduced into Kelly cells. Protein level of MYCN and ASCL1 were analyzed by Western blot. β-actin was used as internal control (c). **(d)** The mRNA expression of *MYCN* after shRNA transduction was measured by qRT-PCR, and *spike-in* was used as internal control. Data are represented as means ± SEM for three biological replicates. The p-value by one-way ANOVA followed by Tukey's multiple comparisons post hoc test are indicated. **(e)** The mRNA expression of *ASCL1* across different cancer cell line were shown in log2 form. Data were analyzed by CCLE database. Data are represented as box plots where the middle line indicates the median, the lower and upper hinges correspond to the first and third quartiles, the lowest datum indicates the minimum, and the highest datum indicates the maximum. Outliners smaller than the 1.5 IQR of the lower quartile or larger than the 1.5 IQR of the upper quartile are shown in dots. **(f)** Heatmap showing list of genes expression after *LMO1* knockdown in SH-SY5Y. Arrows indicate the positions of *LMO1* and *ASCL1* genes. The color scale represents relative expression. **(g,h)** The survival curve analysis for primary neuroblastoma samples in the Versteeg cohort[1] (g) and the Neuroblastoma Research Consortium (NRC) dataset (GSE85047) (h) were done using the R2 database. The samples for which the prognostic data is available were classified into two groups (*ASCL1*-high and low) by the Kaplan Scan method, which calculates the optimum cut-off based on statistical testing. The raw p-value and Bonferroni-corrected p-value are shown.

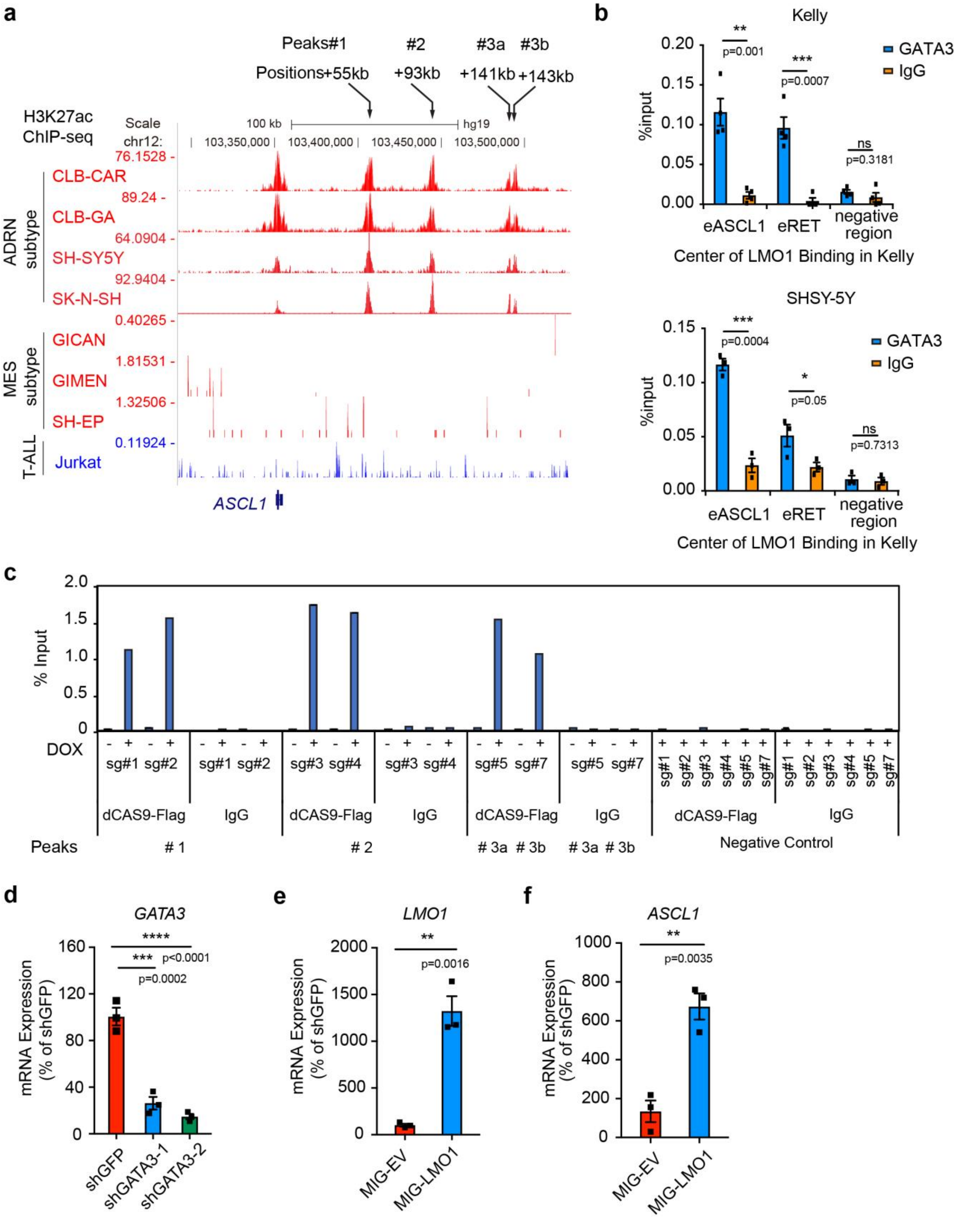

Supplementary Fig. 4: *ASCL* is directly regulated by LMO1 and MYCN in neuroblastoma cells. **(a)** ChIP-seq gene tracks showing the H3K27ac signal at the *ASCL1*

gene locus in four ADRN neuroblastoma cell lines with high level of *LMO1* expression (CLB-CAR, CLB-GA, SH-SY5Y and SKNSH), three MES neuroblastoma cell lines with low level of *LMO1* expression (GICAN, GIMEN and SH-EP) and Jurkat T-ALL cell line. See the Fig. 4a legend for details. **(b)** ChIP-PCR was carried out using an anti-GATA3 or control IgG antibody in Kelly and SH-SY5Y cells. The amount of DNA in the ChIP sample (% of input) at *ASCL1* and *RET* gene locus were shown. As negative controls, which is not associated with either H3K27ac or LMO1, were used. Data are represented as means ± SEM for three biological replicates. The p-values by two-tailed unpaired t test are indicated. **(c)** ChIP-PCR were carried out using an anti-Flag or control IgG in Kelly cells transduced with the Flag-dCas9-KRAB protein and sgRNA treated with or without doxycycline (DOX). The amount of DNA in the ChIP sample (% of input) were shown. Data are represented as mean of technical duplicates. **(d)** The mRNA expression of *GATA3* after shRNA knockdown were measured by qRT-PCR, and *GAPDH* was used as internal control. Data are represented as means ± SEM for three biological replicates. The p-values by one-way ANOVA followed by Tukey's multiple comparisons post hoc test are indicated. **(e, f)** Kelly cells was first transduced with a retrovirus vector encoding the *LMO1* cDNA (MIG-LMO1) or an empty vector (MIG-EV). The cells were then transduced with a shRNA targeting *LMO1* or control (shLUC). The mRNA expression levels for *LMO1* (e) or *ASCL1* (f) were evaluated by qRT-PCR, and *GAPDH* was used as internal control. Data are represented as means ± SEM for three biological replicates. The p-values by two-tailed unpaired t test are indicated.

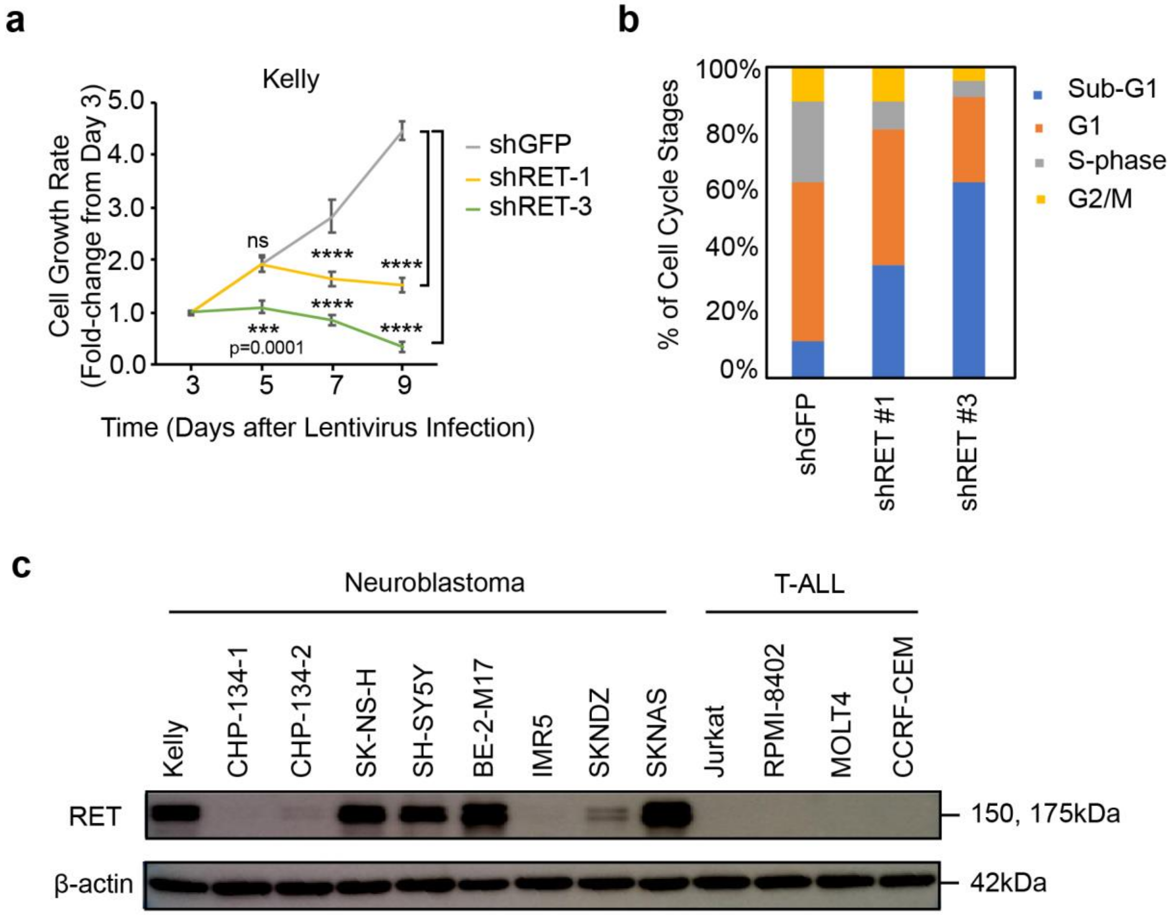

Supplementary Fig. 5: *RET* is directly regulated by LMO1 in neuroblastoma cells. (a) Cell viability was measured after 3, 5, 7 and 9 days of lentiviral transduction of shRNA. The growth rate (fold-change) over 9 days compared to day 3 was indicated (n=3 per group). Values represent means ± standard deviation (SD) for technical replicates. The p-values by two-way ANOVA (repeated measurements) followed by Tukey's multiple comparisons post hoc test are indicated. ****, p-value<0.0001. ns, not significant. (**b)** shRNAs targeting *RET* and control (*GFP*) were transduced by lentivirus infection into Kelly. Approximately one million cells were counted and fixed at 5 days after infection. The cells stained with PI were subjected to flow cytometry analysis. The percentage of cells in each cell cycle phase were shown. **(c)** Protein expression of RET in a panel of neuroblastoma and T-ALL cell lines were analyzed by Western blot. β-actin was used as an internal control.

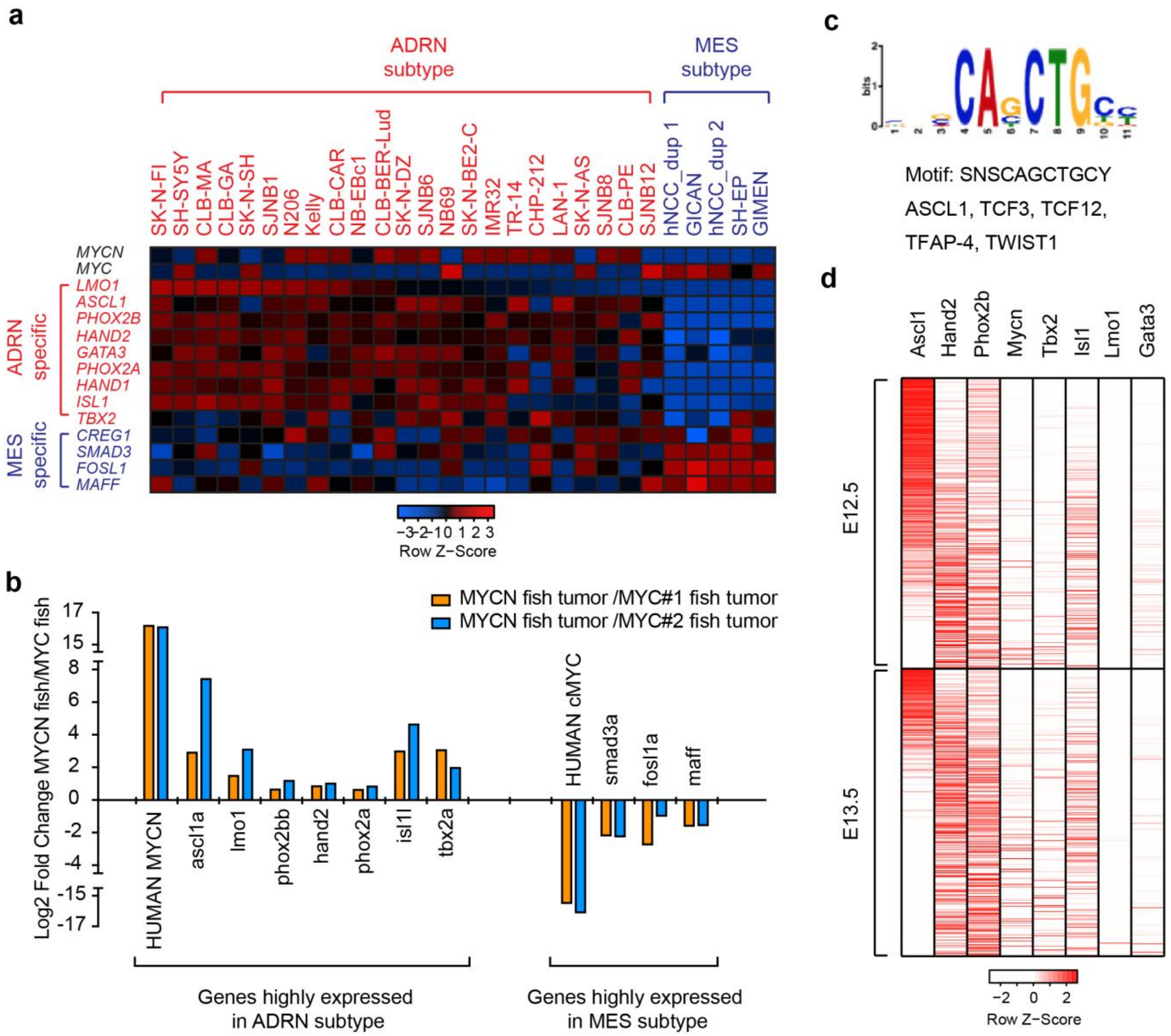

Supplementary Fig. 6: *ASCL1* is a member of CRC in the ADRN subtype of neuroblastoma. (a) Heatmap was generated using global normalization to Log2 TPM. Heatmap image represents gene expression of *LMO1* along with transcription factor genes that are dominantly expressed in the ADRN subtype of neuroblastoma (*MYCN*, *ASCL1*, *PHOX2B*, *HAND2*, *GATA3*, *PHOX2A*, *HAND1*, *ISL1* and *TBX2*) or MES subtype of neuroblastoma (*MYC*, *SMAD3*, *FOSL1* and *MAFF*). Neuroblastoma cell lines were ordered based on expression of *LMO1* from high to low. The color scale represents row z-scores. **(b)** RNA-seq analysis for one *MYCN*-induced zebrafish neuroblastoma and two *c-MYC*-induced zebrafish neuroblastoma (#1 and 2) were carried out in our previous study[2]. Expressions of zebrafish orthologues of transcription factor genes were analyzed. Log_2 fold change values between *c-MYC*-induced zebrafish tumors and *MYCN*-induced tumors were shown. **(c)** DNA binding motif analysis were performed with the ASCL1-bound peaks called by MACS2 (P<1e-4), 500 bps in total were retrieved from the peak summits (the highest point of each peak). Enriched motifs were analyzed using the MEME-chip package from

MEME-Suite. **(d)** The single cell RNA-seq data was reported by Furlan et al.[3] for mouse embryo at stage E12.5 and E13.5. Heatmap was generated using global normalization to Log2 TPM. Heatmap image represents gene expression of *Ascl1* along with transcription factor genes that are dominantly expressed in the ADRN subtype of neuroblastoma (*Ascl1, Hand2*, *Phox2b, Mycn*, *Tbx2, Isl1, Lmo1* and *Gata3*). Singles cells were ordered based on expression of *Ascl1* from high to low. The color scale represents row z-scores.

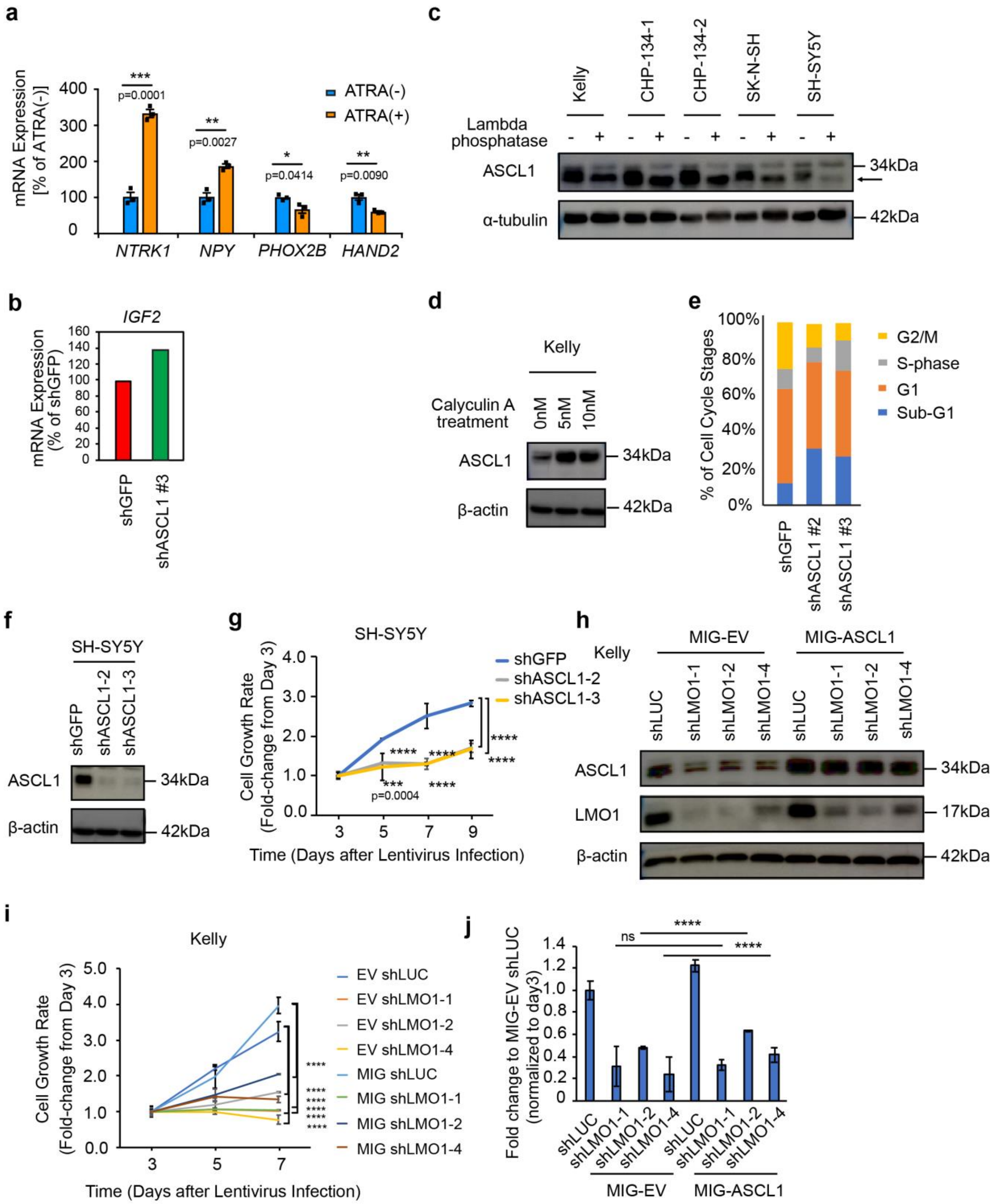

Supplementary Fig. 7: ASCL1 is required for cell growth and regulates neural differentiation status. (a) Kelly cells were treated with or without all-trans retinoic acid (ATRA: 10uM) for seven days. The mRNA expression levels for *NTRK1, NPY, SYP, PHOX2B, HAND2* and *GAPDH* (internal control) were evaluated by qRT-PCR. Gene

expression was normalized to *GAPDH,* calculated as a percent relative to untreated control. Data are represented as means ± SEM for three biological replicates. The p-values by two-tailed unpaired t test are indicated. **(b)** The mRNA expression of *IGF2* after *ASCL1* knockdown were measured by qRT-PCR. Data are represented as mean of technical duplicates. **(c)** Protein expression levels of ASCL1 were analyzed with or without lambda phosphatase treatment in different neuroblastoma cell lines. α-tubulin was used as an internal control. The arrow indicates the dephosphorylated form. **(d)** Protein expressions of ASCL1 were analyzed after calyculin A treatment for 30 min at different concentration 0, 5 and 10nM. **(e)** shRNAs targeting *ASCL1* and control (*GFP)* were transduced by lentivirus infection into Kelly. Approximately one million cells were counted and fixed at 5 days after infection. The cells stained with PI were subjected to flow cytometry analysis. The percentage of cells in each cell cycle phase were shown. **(f)** SH-SY5Y cells were transduced with independent shRNAs targeting *ASCL1* (shASCL1 # 2 and 3) as well as a control shRNA targeting *GFP* (shGFP) by lentivirus infection. Whole cell protein extract was harvested after 3 days of virus infection and subjected to Western blot analysis using antibodies specific for ASCL1 or β-actin (internal control). **(g)** Cell viability was measured after 3, 5, 7 and 9 days of lentiviral transduction of shRNA. The growth rate (fold-change) over 9 days compared to day 3 was indicated (n=3 per group). **(h)** Kelly cells were firstly transduced with *ASCL1* by retrovirus infection (MSCV-ASCL1-GFP) or an empty vector (MSCV-GFP). Independent shRNAs targeting *LMO1* (shLMO1 #1, 2 and 4) as well as a control shRNA targeting *LUC* (shLUC) were then transduced by lentivirus infection. Whole cell protein extract was harvested after 3 days of lentivirus infection and were subjected to Western blot analysis using antibodies specific for LMO1, ASCL1 or β-actin (internal control). **(i)** Cell viability was measured after 3, 5 and 7 days of lentiviral transduction of shRNA. The growth rate (fold-change) over 7 days compared to day 3 was indicated (n=3 per group). **(j)** Cell viability on day 7 were shown in bar-chart. Data are presented as the fold-change of the shGFP control (n=3 per group). Values represent means ± SD for technical triplicates in **g**, **i** and **j**. The p-value by two-way ANOVA (repeated measurements) followed by Tukey's multiple comparisons post hoc test are indicated in **g**, **i** and **j**. ****, p-value<0.0001.

Supplemental Fig. 8: Uncropped images

Figure 1a

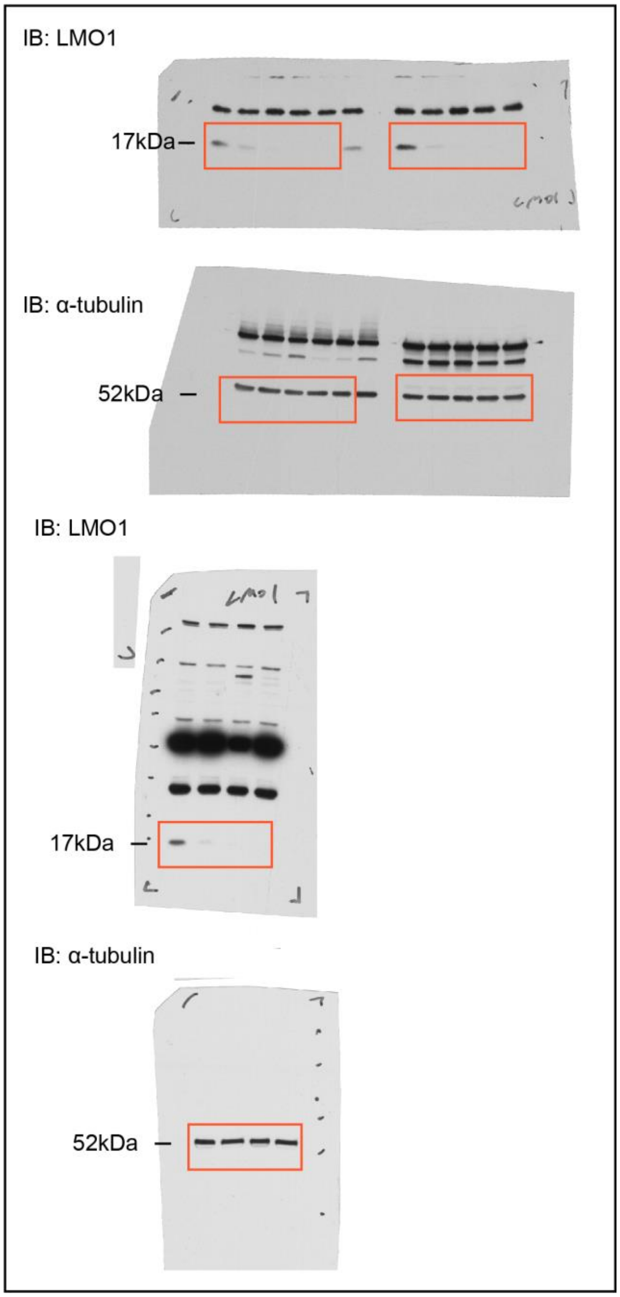

Figure 3b

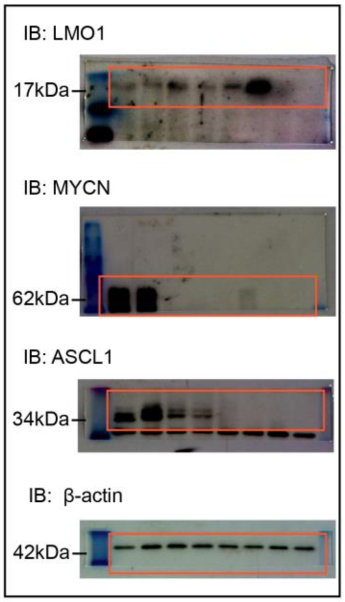

Figure 4b

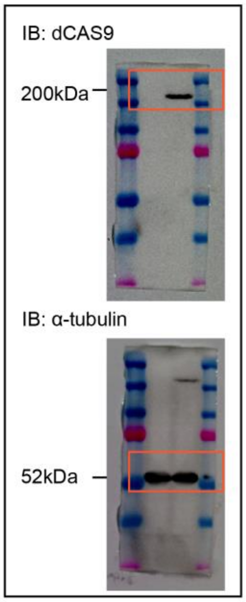

Figure 4c

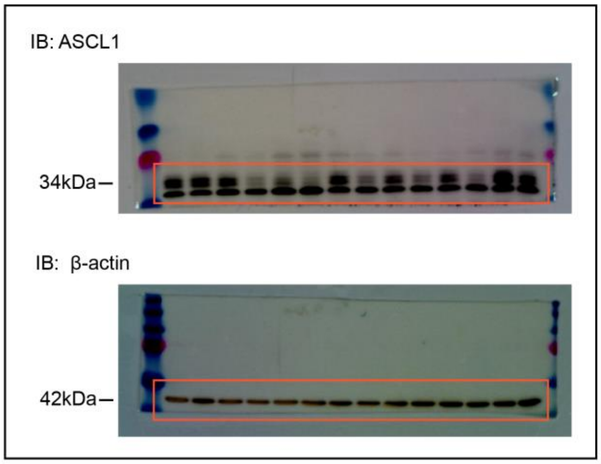

Figure 4f

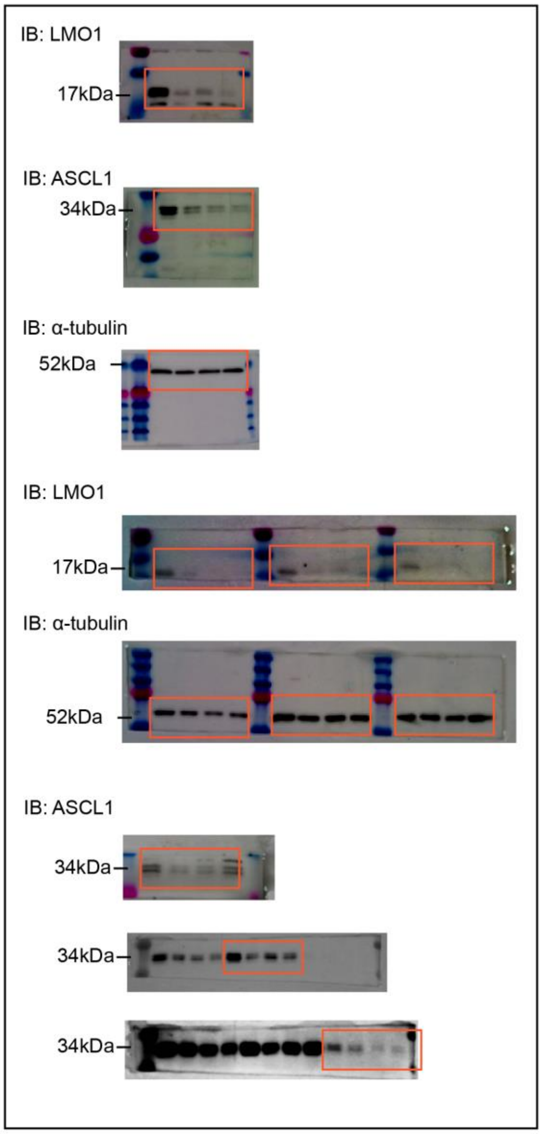

Figure 5c

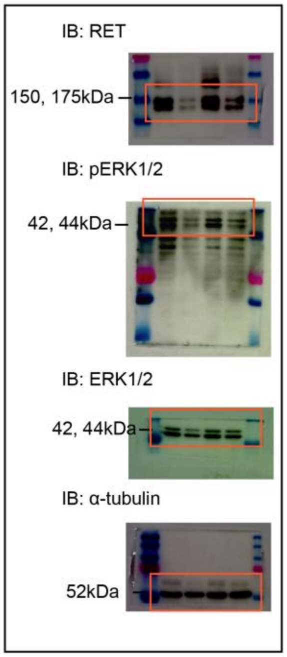

Figure 5c

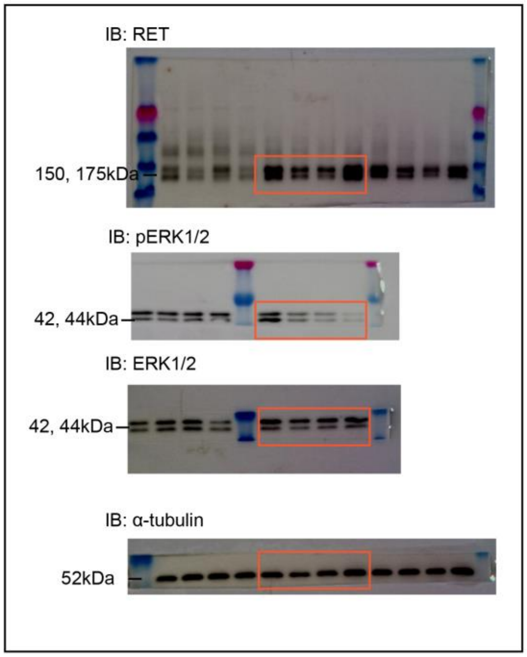

Figure 5e

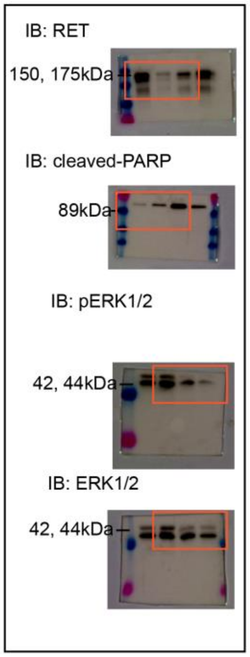

Supplementary Figure 2c

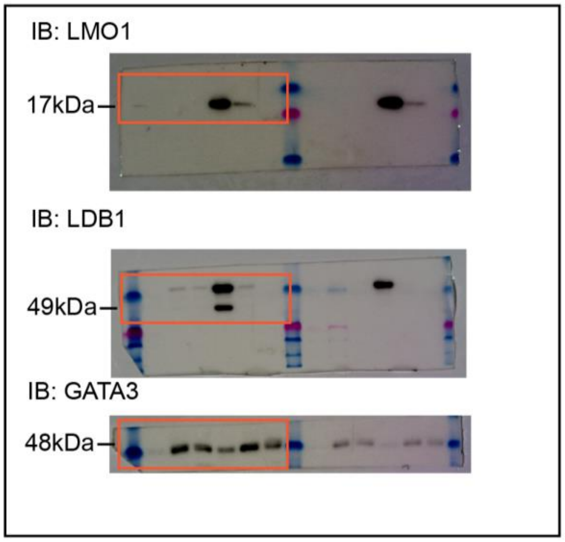

Supplementary Figure 2d

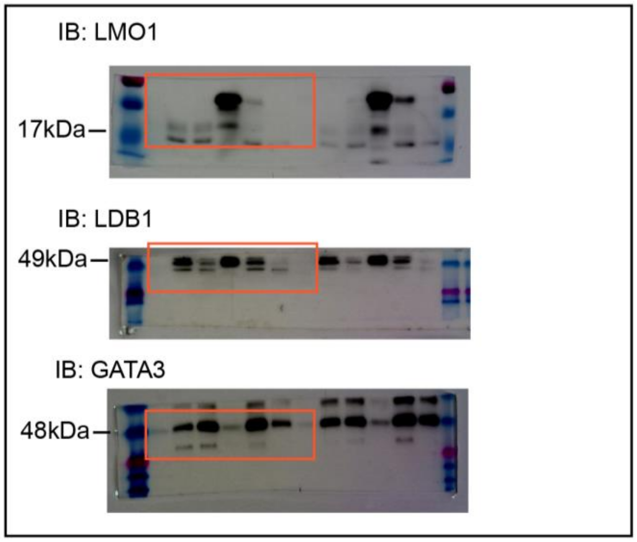

Supplementary Figure 3c

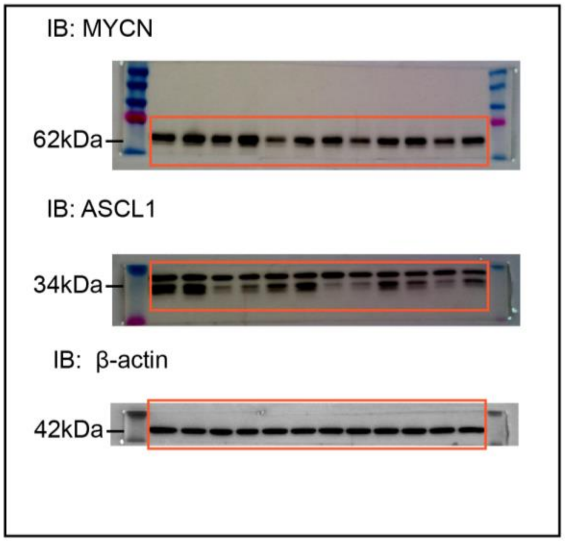

Supplementary Figure 5c

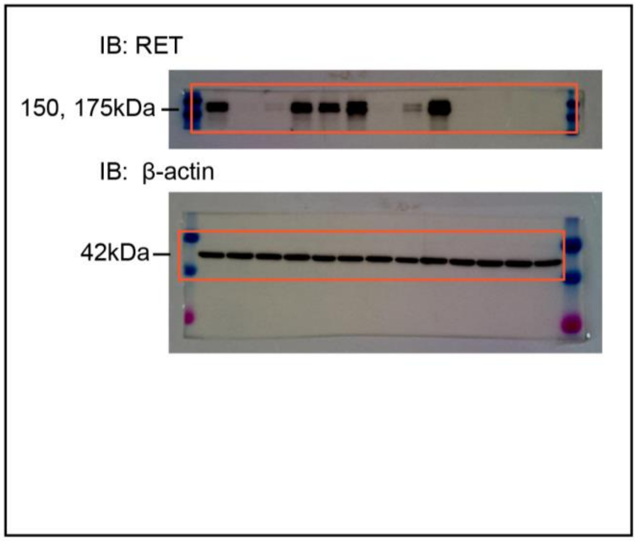

Supplementary Figure 7c

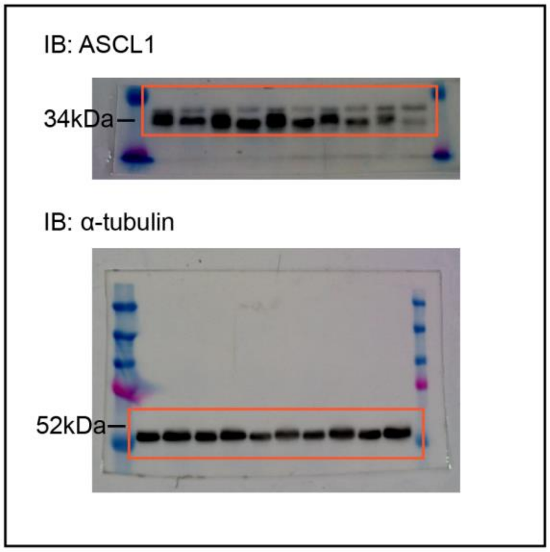

Supplementary Figure 7d

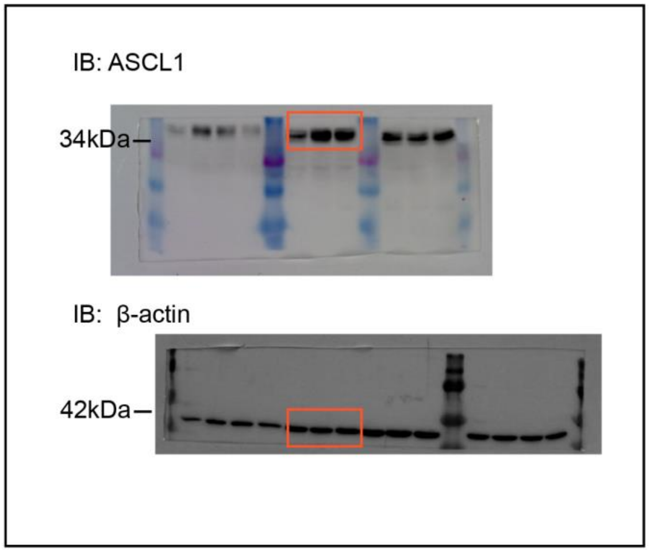

Supplementary Figure 7d

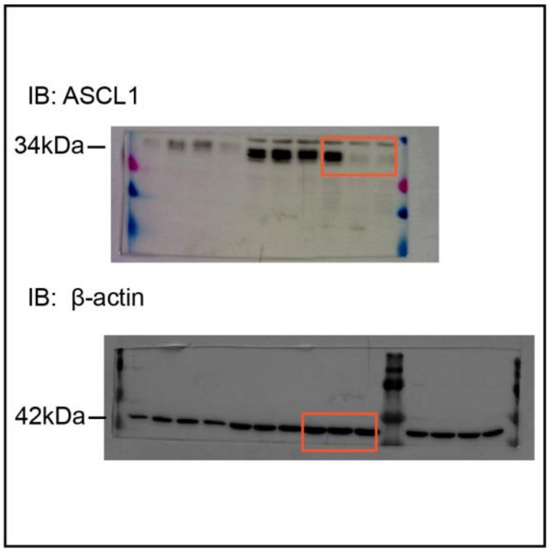

Supplementary Figure 7h

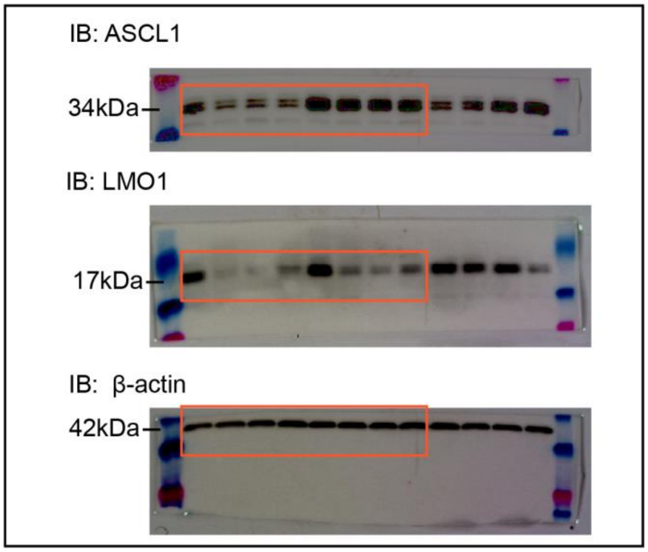

Supplementary Methods

Cell cycle analysis

Approximately 1 million cells were counted, washed with PBS and fixed with 70% ethanol at 4°C for 1 hour. The cells were washed with PBS, treated with 50uL RNase H (final concentration at 2mg/mL) for 30 min at 37°C, and incubated with 200uL of 50ug/mL propidium iodide (PI). The cells were then subjected to flow cytometry analysis using the BD LSRII (BD Biosciences). The percentage of cells in each cell cycle phase was measured.

Motif analysis

Based on the ChIP-seq peaks called by MACS14, 500 bps in total were retrieved from the peak summits. Enriched motifs were analyzed using the MEME-chip package from MEME-Suite [4] using the Homo sapiens Comprehensive Model Collection database (HOCOMOCO) v11 and the TRANSFAC motif databases.

Microarray analysis in SH-SY5Y cells

Total RNA was extracted from SH-SY5Y cells transduced with a control shRNA (shGFP) or LMO1 shRNA (shLMO#2) using the QIAzol lysis reagent (Qiagen) and cleaned using the RNeasy kit (Qiagen). A total of 4 RNA samples (2 controls and 2 knockdowns; biological duplicates) were used. Genome-wide RNA expression analysis was performed using the HG U133 plus 2.0 microarray chip (Affymetrix, Santa Clara, CA) at the Dana-Farber Cancer Institute, Boston. Array signals were normalized using RMA (http://rmaexpress.bmbolstad.com/). Differential gene expression was determined using a moderated t-test in the “limma” package (http://bioinf.wehi.edu.au/limma/) from Bioconductor (www.bioconductor.org).

Gene set enrichment analysis (GSEA)

GSEA[5] analysis of the normalized genes was performed using the log2 ratio of classes metric to rank genes for the comparison between control and *LMO1* knockdown samples in SH-SY5Y cells. The direct target genes regulated by *LMO1* in Kelly cells were first defined by ChIP-seq based on peak calling and by RNA-seq based on a significant gene expression change upon shRNA knockdown (absolute log2-fold-change ≥ or ≤1; p-value <0.05). The lists of upregulated genes and downregulated genes were used as gene sets.

Lamda phosphatase treatment

$1X10^6$ cells were lysed with RIPA buffer in the presence of protease inhibitor (Roche). A total of 1uL lambda phosphatase (NEB, P0753S) and 1X NEB buffer (final concentration) for protein metallophosphatases (PMP) and 1mM $MnCl_2$ (final concentration) were added to lysate to make a total reaction volume of 50uL and incubated at 30 degree for 30 minutes. Lysate was then mixed with Laemmli sample buffer (Bio-Rad) with 10% beta-mercaptoethanol and boiled for 10 min at 95 °C for western blot analysis.

Calyculin A treatment

A total of 800,000 cells were seeded one day before in 6-well plates. The cells were then treated with Calyculin A (Cell Signaling, #9902) at different concentration for 30 min and then collected for Western blot analysis.

Immunoprecipitation (IP) analysis

The cells were lysed in Lysis Buffer [50mM Tris (pH 7.5), 1mM EDTA, 150mM NaCl, 0.1% SDS, 0.5% NP40, 10% glycerol and protease inhibitor cocktail (Roche)]. Anti-FLAG M2 affinity agarose were washed with washing buffer [50mM Tris (pH 7.5), 1mM EDTA, 150mM NaCl, 0.1% SDS, 0.5% NP40, 10% glycerol] and incubated with cell lysate overnight. A portion of cell lysate was saved as input. On the following day, beads were washed twice with washing buffer. Proteins were eluted with the FLAG peptide in the FLAG buffer [50mM Tris (pH 7.5), 1mM EDTA, 150mM NaCl, 0.1% SDS, 0.5% NP40, 10% glycerol and protease inhibitor cocktail (Roche)] by agitating at 4 degree for 30 min. Elutes and input samples were supplemented with the Laemmli sample buffer (Bio-Rad) with 10% beta-mercaptoethanol and boiled for 10 min at 95 °C. Protein samples were resolved by SDS-PAGE by standard methods. Proteins were visualized by enhanced chemiluminescence (Thermo).

***LMO1* overexpression experiment in 293T cells**

The *LMO1* cDNA (CDS region of LMO1 transcript, NM_001270428.1) was reverse-transcribed and amplified from the RNA extracted from Kelly cells and then cloned into the pOZ-C/N vector. A total of 100ng pOZ-C/N vector or pOZ-C/N empty vector were co-transfected into 293T cells (200,000 cells) seeded into 6-well plates 24 hours before transfection with 100ng of each pCS2 plasmids coding LDB1 and GATA3 using FuGENE 6 reagent (Roche) and Opti-MEM. Cells were collected 48 hours after transfection and subjected to IP analysis.

***LMO1* overexpression experiment in Kelly cells**

The *LMO1* cDNA (CDS region of LMO1 transcript, NM_001270428.1) was reverse-transcribed and amplified from the RNA extracted from Kelly cells and then cloned into the pOZ-C/N retrovirus vector. Retroviral vector (1,000ng) was co-transfected into 293T cells (200,000 cells) seeded into 6-well plates 24 hours before transfection with the packaging plasmid GAG (250ng) and the envelope plasmid ENV (250ng) using FuGENE 6 reagent (Roche) and Opti-MEM. Approximately 500,000 cells were transduced with 1 mL retrovirus media in the presence of polybrene (8 ug/ml: Millipore) for 2 hours incubation followed by toping up of 1 mL fresh medium. The cells that express CD25 (selection marker) were then sorted by flow cytometry using the BD FACSAria II (BD Biosciences) after stained with an anti-CD25 antibody (Biolengend). Kelly cell lines stably transduced with empty pOZ-C/N vector were used as control.

***ASCL1* overexpression and rescue study**

The *ASCL1* cDNA (CDS region of ASCL1 transcript) was reverse-transcribed and amplified from the RNA extracted from Kelly cells and then cloned into the pMSCV-IRES-GFP retrovirus vector. Retroviral vector (1,000ng) was co-transfected into 293T cells (200,000 cells) seeded into 6-well plates 24 hours before transfection with the packaging plasmid pMD-MLV (250ng) and the envelope plasmid VSV-G (250ng) using FuGENE 6 reagent (Roche) and Opti-MEM. Approximately 500,000 cells were transduced with 1 mL retrovirus media in the presence of polybrene (8 ug/ml: Millipore) for 2 hours incubation followed by toping up of 1 mL fresh medium. The cells that express GFP (selection marker) were then sorted by flow cytometry using the BD FACSAria II (BD Biosciences). Kelly cell lines stably

transduced with pMSCV-IRES-GFP ASCL1 or pMSCV-IRES-GFP empty vector were then transduced with individual shRNAs targeting LMO1 by lentivirus infection.

Statistics and Reproducibility

All the statistical analyses were done in GraphPad Prism software. A p-value less than 0.05 was considered statistically significant. The details of methods used can be found in each supplementary figure legend. Experiments in Supplementary Fig 1a, 2c, 2d, 3d, 4b, 4d, 4e, 4f and 7a were repeated three or more times. Experiments in Supplementary Fig 3c, 4c, 5a, 5b and 7e were repeated two times.

Supplementary References

1. Molenaar JJ*, et al.* Sequencing of neuroblastoma identifies chromothripsis and defects in neuritogenesis genes. *Nature* **483**, 589-593 (2012).
2. Zimmerman MW*, et al.* MYC Drives a Subset of High-Risk Pediatric Neuroblastomas and Is Activated through Mechanisms Including Enhancer Hijacking and Focal Enhancer Amplification. *Cancer Discov* **8**, 320-335 (2018).
3. Furlan A*, et al.* Multipotent peripheral glial cells generate neuroendocrine cells of the adrenal medulla. *Science* **357**, (2017).
4. Grant CE, Bailey TL, Noble WS. FIMO: scanning for occurrences of a given motif. *Bioinformatics* **27**, 1017-1018 (2011).
5. Subramanian A*, et al.* Gene set enrichment analysis: a knowledge-based approach for interpreting genome-wide expression profiles. *Proc Natl Acad Sci U S A* **102**, 15545-15550 (2005).
