# Supplementary material for: ASCL1 is a MYCN- and LMO1-dependent member of the adrenergic neuroblastoma core regulatory circuitry
Source: Nat Commun. 2019 Dec 9;10:5622. doi: 10.1038/s41467-019-13515-5 (PMC6901540; doi:10.1038/s41467-019-13515-5)
Supplement: Supplementary file 2 — Description of Additional Supplementary Files [file 41467_2019_13515_MOESM2_ESM.pdf]

Description of Additional Supplementary Files

File Name: Supplementary Data 1
Description: List of genes significantly regulated after LMO1 knockdown

File Name: Supplementary Data 2
Description: List of direct target genes

File Name: Supplementary Data 3
Description: shRNA sequences

File Name: Supplementary Data 4
Description: qRT-PCR primer sequences

File Name: Supplementary Data 5
Description: sgRNA target sequences

File Name: Supplementary Data 6
Description: ChIP-PCR primer sequences

File Name: Supplementary Data 7
Description: Dataset accession numbers
