# Supplementary material for: ASCL1 is a MYCN- and LMO1-dependent member of the adrenergic neuroblastoma core regulatory circuitry
Source: Nat Commun. 2019 Dec 9;10:5622. doi: 10.1038/s41467-019-13515-5 (PMC6901540; doi:10.1038/s41467-019-13515-5)
Supplement: Supplementary file 10 — Reporting Summary [file 41467_2019_13515_MOESM10_ESM.pdf]

nature research

Corresponding author(s): Takaomi Sanda, A Thomas Look

Last updated by author(s): Nov 6, 2019

Reporting Summary

Nature Research wishes to improve the reproducibility of the work that we publish. This form provides structure for consistency and transparency in reporting. For further information on Nature Research policies, see Authors & Referees and the Editorial Policy Checklist.

Statistics

For all statistical analyses, confirm that the following items are present in the figure legend, table legend, main text, or Methods section.

n/a	Confirmed	
☐	☒	The exact sample size (*n*) for each experimental group/condition, given as a discrete number and unit of measurement
☐	☒	A statement on whether measurements were taken from distinct samples or whether the same sample was measured repeatedly
☐	☒	The statistical test(s) used AND whether they are one- or two-sided<br>*Only common tests should be described solely by name; describe more complex techniques in the Methods section.*
☒	☐	A description of all covariates tested
☐	☒	A description of any assumptions or corrections, such as tests of normality and adjustment for multiple comparisons
☐	☒	A full description of the statistical parameters including central tendency (e.g. means) or other basic estimates (e.g. regression coefficient) AND variation (e.g. standard deviation) or associated estimates of uncertainty (e.g. confidence intervals)
☐	☒	For null hypothesis testing, the test statistic (e.g. *F*, *t*, *r*) with confidence intervals, effect sizes, degrees of freedom and *P* value noted<br>*Give P values as exact values whenever suitable.*
☒	☐	For Bayesian analysis, information on the choice of priors and Markov chain Monte Carlo settings
☒	☐	For hierarchical and complex designs, identification of the appropriate level for tests and full reporting of outcomes
☒	☐	Estimates of effect sizes (e.g. Cohen's *d*, Pearson's *r*), indicating how they were calculated

Our web collection on statistics for biologists contains articles on many of the points above.

Software and code

Policy information about availability of computer code

Data collection	Not applicable.
Data analysis	All ChIP-seq reads were mapped to the hg19 human reference genome using bowtie2 with default parameters. Duplicates were removed from aligned ChIP-seq reads using the samtools rmdup package. ChIP-seq peaks were called with MACS14 software version 1.4.2 The output bedgraph were normalized subtracting corresponding background using MACS2 bdgcmp. LMO1 peaks (P<1e-4) and ASCL1 peaks (P<1e-9) called by MACS14 were selected and filtered for non-promoter binding peaks based on annotatePeaks from the HOMER package. Co-occurrence heatmap and metagene were plotted using deepTools version 3.1.2. Each of the signal matrix were calculated using bigwig output generated using UCSC bedGraphToBigWig of each ChIP-seq sample by deepTools computerMatrix. The signal matrix were then used to plot heatmap using deepTools plotHeatmap package.<br><br>H3K7ac peaks were called using MACS2 version 1.4.2 (q-value<5e-2 for narrow region and q-value < 1e-1 for broad region) with –keep-dup=1 and --broad. Enhancers were identified using ROSE v0.1 by merging H3K27ac peaks within 12.5 kb between each other, excluded those were fully contained within +/-2kb from TSS and ranked them along the x-axis based on H3K27ac enrichment plotted on the y-axis (https://bitbucket.org/youngcomputation/rose).<br><br>RNA-seq reads were aligned to the hg19 human reference genome using STAR 2.5.2a with outFilterMultimapNmax set to 1. Total mapped reads were quantified using htseq-count version 0.6.1, and count tables were generated based on Ensembl hg19 gene annotation gtf files. Differential expression analysis was conducted using the Bioconductor package DESeq2 version 1.12.4. Gene expression for each neuroblastoma cell lines was estimated in transcripts per million (TPM) using Kallisto software version 0.43.1. All RNA-Seq data were normalized using the Sleuth R package from Patcher lab. Selected gene expression from sleuth normalized TPM were used to generate heatmap using web-based heatmap tools, heatmapper.<br><br>GO analysis was performed using the Enrichr tool (http://amp.pharm.mssm.edu/Enrichr/).

For manuscripts utilizing custom algorithms or software that are central to the research but not yet described in published literature, software must be made available to editors/reviewers. We strongly encourage code deposition in a community repository (e.g. GitHub). See the Nature Research guidelines for submitting code & software for further information.

Data

Policy information about availability of data

All manuscripts must include a data availability statement. This statement should provide the following information, where applicable:

- Accession codes, unique identifiers, or web links for publicly available datasets
- A list of figures that have associated raw data
- A description of any restrictions on data availability

ChIP-seq dataset for LMO1 and ASCL1 in Kelly cells have been deposited in the GEO database (GSE120074).
ChIP-seq datasets of PHOX2B, HAND2, GATA3, MYCN, ISL1 and TBX2, H3K27ac and H3K4me1 in Kelly cells were obtained from the GEO database (GSE94824 and GSE62726).
ChIP-seq datasets of H3K27ac in various neuroblastoma cell lines were obtained from the GEO database (GSE86672 and GSE90683).
ChIP-seq datasets of LMO1, GATA3, H3K27ac and H3K4me1 in Jurkat cells were obtained from the GEO database (GSE94391, GSE68976 and GSE50622, GSE119439).
RNA-seq dataset for Kelly cells after LMO1 knockdown and ASCL1 knockdown were deposited in the GEO database (GSE132760).
Microarray dataset for SH-SY5Y cells after LMO1 knockdown were deposited in the GEO database (GSE130747).
RNA-seq dataset for Jurkat after LMO1 knockdown has been reported by us and deposited in the GEO database (GSE97514).
RNA-seq datasets for zebrafish neuroblastoma samples reported by us were obtained from the GEO database (GSE107518).
RNA-seq dataset for various neuroblastoma cell lines were obtained from the GEO database (GSE90683).
Single cell sequencing dataset for mouse neuronal cells were obtained from the GEO database (GSE99933).
The microarray datasets for primary neuroblastoma cases reported by the Seqc Maqc-Iii Consortium (SEQC) and Kocak et al., were analyzed by the R2 database (http://hgserver1.amc.nl/cgi-bin/r2/main.cgi).
The cancer cell line dataset is derived from CCLE database (https://portals.broadinstitute.org/ccle).

Field-specific reporting

Please select the one below that is the best fit for your research. If you are not sure, read the appropriate sections before making your selection.

☒ Life sciences ☐ Behavioural & social sciences ☐ Ecological, evolutionary & environmental sciences

For a reference copy of the document with all sections, see nature.com/documents/nr-reporting-summary-flat.pdf

Life sciences study design

All studies must disclose on these points even when the disclosure is negative.

Sample size	For cellular and molecular assays, sample sizes were chosen based on expected phenotypes and previous experience with assay variability.<br><br>All the statistical analysis were done in GraphPad Prism software. A p-value less than 0.05 was considered statistically significant. The details of methods used can be found in each figure legend.<br><br>Experiments in Fig 1a, 1b, 3b, 4b, 4d, 4e, 5b, 5d, 7a, 7b, 7c, 7d, 7e and 7f were repeated three or more times. Experiments in Fig 4c, 4f, 5c, 5e were repeated two times. The numbers of experiments conducted in Supplementary Figures were stated in Supplementary Methods section and figure legends.
Data exclusions	For the analysis of gene expression in primary tumors, the samples for which prognostic data was available were used.<br>No data exclusions were performed for other analyses.
Replication	Each experiment was done in replicated. The numbers were indicated in figure legend.
Randomization	Not applicable.
Blinding	Not applicable.

Reporting for specific materials, systems and methods

We require information from authors about some types of materials, experimental systems and methods used in many studies. Here, indicate whether each material, system or method listed is relevant to your study. If you are not sure if a list item applies to your research, read the appropriate section before selecting a response.

Materials & experimental systems

n/a	Involved in the study	
☐	☒	Antibodies
☐	☒	Eukaryotic cell lines
☒	☐	Palaeontology
☒	☐	Animals and other organisms
☒	☐	Human research participants
☒	☐	Clinical data

Methods

n/a	Involved in the study	
☐	☒	ChIP-seq
☒	☐	Flow cytometry
☒	☐	MRI-based neuroimaging

Antibodies

Antibodies used

LMO1 antibody (Bethyl Laboratories, #A300314A)
ASCL1 antibody (Santa Cruz, #sc-390794)
GATA3 (Thermo, #MA1-028, clone 1A12-1D9)
LDB1(Santa Cruz, #sc-365074)
MYCN (Cell Signaling Technologies, #84406),
control IgG antibody(Santa Cruz, #sc-2025)
cas9 antibody (Cell Signaling, #14697)
neurofilament antibody (Cell Signaling, #2837)
CD25 antibody conjugated with PE (Biolengend, #102007).
FLAG antibody (Sigma, #F1804)
RET (Cell Signaling Technologies, #14299)
ERK (Cell Signaling Technologies, #9102)
phosphor-ERK (Cell Signaling Technologies, #9101)
α-tubulin (Cell Signaling Technologies, #2144)
β-actin (Cell Signaling Technologies, #8457)
HRP-labelled anti-rabbit (Cell Signaling Technologies, #7074)
HRP-labelled anti-mouse (Cell Signalling, #7076P2)

Validation

LMO1 antibody (rabbit source), ASCL1 antibody (mouse source) and RET (rabbit source) were validated for Western blot and ChIP-seq experiments.
Cas9 antibody (mouse source) and LDB1 antibody (mouse source) were validated for Western blot experiment.
CD25 antibody conjugated with PE was validated for Flow cytometery analysis.
FLAG antibody (mouse source) and control IgG (mouse source) were validated for ChIP-PCR experiment.
GATA3 antibody (mouse source) was validated for ChIP-PCR and Western blot experiments.
Neurofilament antibody (mouse source) was validated for immunostaining.
MYCN antibody (rabbit source), α-tubulin antibody (rabbit source), β-actin antibody (rabbit source), ERK antibody (rabbit source) and phospho-ERK antibody (rabbit source) were validated by Western blot experiments.

Eukaryotic cell lines

Policy information about cell lines

Cell line source(s)

All neuroblastoma cell lines (Kelly, SH-SY5Y, CHP-134 and SK-N-SH) were derived from human neuroblastoma patients and have been stocked in the Look and Sanda laboratories, which were originally purchased from ATCC and DSMZ cell banks.

T-ALL cell lines (Jurkat, RPMI-8402, MOLT-4 and CCRF-CEM) are derived from human T-ALL patients and and have been stocked in the Look and Sanda laboratories, which were originally purchased from ATCC and DSMZ cell banks

Authentication

DNA fingerprinting using the PowerPlex 1.2 system (Promega, Madison, WI, USA).

Mycoplasma contamination

All cell lines were regularly tested for mycoplasma contamination to validate that they are negative.

Commonly misidentified lines
(See ICLAC register)

No commonly misidentified lines listed in the ICLAC database were used in this study.

ChIP-seq

Data deposition

☒ Confirm that both raw and final processed data have been deposited in a public database such as GEO.

☒ Confirm that you have deposited or provided access to graph files (e.g. BED files) for the called peaks.

Data access links
May remain private before publication.

Link: https://www.ncbi.nlm.nih.gov/geo/query/acc.cgi?acc=GSE120074
The accession key: oruremoiddujnal

Files in database submission

GSM3392950 Kelly_input_R1_MWZ5393 [Lab: Look]
GSM3392951 Kelly_input_X2_MWZ5393 [Lab: Look]
GSM3392952 Kelly_LMO1_R1_MWZ5393 [Lab: Look]
GSM3392953 Kelly_LMO1_X2_MWZ5393 [Lab: Look]
GSM3462607 Kelly_ASCL1_MWZ5393 [Lab: Look]

Each has a bedgraph and a FASTQ available.

Genome browser session
(e.g. UCSC)

No longer available

Methodology

Replicates

For each experiment, individual P150 plates of Kelly cells were fixed and pooled to achieve a total of 1x10E8 cells per pellet. For each ChIP, 10 µg of antibody was conjugated to 2 mg of M270 epoxy beads and added to 3 ml of sonicated nuclear extract. Duplicates were done ChIP-seq.

Sequencing depth

ChIP-sequencing was performed using Illumina NextSeq 500 single-end 75bp reads.

Read counts for ChIP targets were as follows:
IInput R1 reads: 43219074
Input X2 reads: 150156222
LMO1 R1 reads: 23300668
LMO1 X2 read: 25019476
ASCL1 reads: 20697245

Antibodies

Antibodies were first validated by IP with total protein lysates in Kelly cells. The antibodies used for ChIP are as follows:
LMO1 antibody (Bethyl Laboratories, #A300314A)
ASCL1 antibody (Santa Cruz, #sc-390794)

Peak calling parameters

LMO1 peaks (P<1e-4) and ASCL1 peaks (P<1e-9) called by MACS14 with -m 10,30 --keep-dup=1 -B. The output bedgraph were merge and normalized by subtracting corresponding background using MACS2 bdgcmp.

Data quality

Data quality was assessed using MACS by comparing peak enrichment over input controls with a p cutoff value of 1e-9.

Software

All ChIP-seq reads were mapped to the hg19 human reference genome using bowtie2 with default parameters. Duplicates were removed from aligned ChIP-seq reads using the samtools rmdup package.

For visualization, bedGraph file was generated by comparing the treatment to control signal generated in peak calling using -B parameter in MACS14. Utilizing bdgcmp software from MACS2 package, the signal to control were normalize using subtract mode and signal below 0.1 were filtered.

Heatmaps of ChIP-Seq signal in these regions were made by measuring signal in bigWig file using computeMatrix package from deepTools version 3.1. The heatmap was generated using plotHeatmap package from deepTools referencing on 3000 bps both ends from the summit of the peaks.

Enhancer ranking analysis
Enhancers were regions of H3K27ac ChIP-seq enrichment regions. H3K7ac peaks were called using MACS2 version 1.4.2 (q-value<5e-2 for narrow region and q-value < 1e-1 for broad region) with –keep-dup=1 and --broad. Enhancers were identified using ROSE v0.1 by merging H3K27ac peaks within 12.5 kb between each other, excluded those were fully contained within +/-2kb from TSS and ranked them along the x-axis based on H3K27ac enrichment plotted on the y-axis (https://bitbucket.org/youngcomputation/rose). All enhancers were assigned responsible to the nearest RefSeq genes. The percentile of ASCL1 enhancer was measured by ordering the ranking index and calculating the top ranking of ASCL1 enhancer among all other enhancers in each cell line.
